# The apple C2H2-type zinc finger transcription factor MdZAT10 positively regulates JA-induced leaf senescence by interacting with MdBT2

**DOI:** 10.1038/s41438-021-00593-0

**Published:** 2021-07-01

**Authors:** Kuo Yang, Jian-Ping An, Chong-Yang Li, Xue-Na Shen, Ya-Jing Liu, Da-Ru Wang, Xing-Long Ji, Yu-Jin Hao, Chun-Xiang You

**Affiliations:** 1 National Key Laboratory of Crop Biology, Shandong Collaborative Innovation Center of Fruit & Vegetable Quality and Efficient Production, College of Horticulture Science and Engineering, Shandong Agricultural University, Tai-An, Shandong 271018 China; 2 National Key Laboratory of Crop Biology, College of Life Science, Shandong Agricultural University, Tai-An, Shandong 271018 China

**Keywords:** Jasmonic acid, Abiotic

## Abstract

Jasmonic acid (JA) plays an important role in regulating leaf senescence. However, the molecular mechanisms of leaf senescence in apple (*Malus domestica*) remain elusive. In this study, we found that MdZAT10, a C2H2-type zinc finger transcription factor (TF) in apple, markedly accelerates leaf senescence and increases the expression of senescence-related genes. To explore how MdZAT10 promotes leaf senescence, we carried out liquid chromatography/mass spectrometry screening. We found that MdABI5 physically interacts with MdZAT10. MdABI5, an important positive regulator of leaf senescence, significantly accelerated leaf senescence in apple. MdZAT10 was found to enhance the transcriptional activity of MdABI5 for *MdNYC1* and *MdNYE1*, thus accelerating leaf senescence. In addition, we found that *MdZAT10* expression was induced by methyl jasmonate (MeJA), which accelerated JA-induced leaf senescence. We also found that the JA-responsive protein MdBT2 directly interacts with MdZAT10 and reduces its protein stability through ubiquitination and degradation, thereby delaying MdZAT10-mediated leaf senescence. Taken together, our results provide new insight into the mechanisms by which MdZAT10 positively regulates JA-induced leaf senescence in apple.

## Introduction

Plant leaf senescence, the last stage of leaf development, is accompanied by a series of physiological and biochemical changes, including the degradation of intracellular organelles and hydrolysis of macromolecules for the relocation of nutrients and energy into newly developing tissues or storage organs^1^. It is important to understand how plants regulate the senescence process to prevent major yield losses in agriculture. The leaf senescence process can be triggered and promoted by unfavorable environmental cues, including extended darkness, drought, and pathogen attack^2–4^, and by endogenous factors such as age, developmental stage, and plant hormones^5,6^. Leaf senescence inhibits photosynthetic capacity and thus decreases crop quality and yield^7,8^. Therefore, delaying leaf senescence offers potential economic benefits^7^.

Plant hormones are known to affect the timing of leaf senescence. Hormones such as abscisic acid (ABA), jasmonic acid, ethylene (ET), and salicylic acid (SA) accelerate the leaf senescence process, whereas auxin, cytokinins (CKs), and gibberellic acid (GA) delay leaf senescence^9^. JA is a lipid-derived phytohormone that is ubiquitous in the plant kingdom and plays essential roles in the regulation of multiple physiological processes in plants, including root growth, leaf senescence, and the response to wounding and pathogens^10–13^. In *Arabidopsis*, the endogenous JA content is higher in senescent leaves than in nonsenescent leaves^14^. Consistent with this increased JA content, several genes involved in the JA biosynthesis pathway, such as *LIPOXYGENASE 1/3/4* (*LOX1/3/4*) and *ALLENE OXIDE CYCLASE 1* (*AOC1*), are also markedly upregulated during leaf senescence^14^. In response to JA, the JASMONATE ZIM-DOMAIN (JAZ) proteins interact with CORONATINE INSENSITIVE1 (COI1), a component of the SCF^COI1^ complex^15,16^. The JAZ proteins are then degraded by the 26S proteasome, thereby releasing downstream JA-responsive genes such as the bHLH transcription factor MYC2^16,17^. MYC2 positively regulates JA-induced leaf senescence by directly activating the expression of *SENESCENCE-ASSOCIATED GENE 29* (*SAG29*), and MYC2 interacts with the bHLH subgroup IIId TFs bHLH03, 13, 14, and 17, which antagonistically regulate leaf senescence^18^.

A large number of genetic and transcriptome studies have shown that TFs regulate the leaf senescence process^10,19^. Some TFs play critical roles in leaf senescence regulatory networks; these TFs include members of the bHLH, NAC, MYB, WRKY, bZIP, C2H2-type zinc finger, and AP2⁄EREBP families^19,20^. C2H2-type zincfinger proteins (ZFPs) are a large family of transcriptional regulators in plants^21^. Various C2H2-type zinc finger TFs are known to be involved in plant development and stress responses^22,23^. Most ZFPs contain one to four highly conserved QALGGH motifs^24^. In addition, a few ZFPs contain C-terminal ERF-associated amphiphilic repression (EAR) motifs, which function as transcriptional repressors^25,26^. Several members of the C2H2-type zinc finger TF family were found to be up- or downregulated during natural leaf senescence^19^, indicating that C2H2-type zinc finger TFs may participate in leaf senescence. Arabidopsis zinc-finger protein 2 (AZF2), a C2H2-type zinc finger TF, positively regulates age-triggered leaf senescence^27^.

ABSCISIC ACID-INSENSITIVE5 (ABI5), a basic leucine zipper (bZIP)-type TF, positively regulates ABA signaling and participates in seed germination, abiotic stress tolerance, and leaf senescence^4,28,29^. Previous studies have revealed that ABI5 modulates leaf senescence by transcriptional regulation. ABI5 positively regulates dark-induced leaf senescence by directly repressing the expression of *ABA-response protein* (*ABR*)^30^ and activating the expression of the chlorophyll degradation genes *NON-YELLOW COLORING1* (*NYC1*) and *STAY-GREEN 1* (*SGR1/NYE1*)^4^. The bHLH TFs PHYTOCHROME-INTERACTING FACTORS 4/5 (PIF4/5) directly activate *ABI5* during dark-induced senescence^4^. In rice (*Oryza sativa*), ONAC054 directly activates *OsABI5* in response to leaf senescence^31^. A recent study found that MdABI5 is involved in ABA-induced leaf senescence^32^. These findings indicate that ABI5 plays a crucial role in the leaf senescence process.

Previous studies have identified several genes that promote or delay leaf senescence in apple^33–35^. In this study, we identified a C2H2-type zinc finger TF, MdZAT10, and demonstrated that it positively regulates leaf senescence in apple (*Malus domestica*). Further experiments showed that MdZAT10 interacts with MdABI5 and accelerates the MdABI5-mediated leaf senescence process. MdZAT10 was also found to accelerate JA-induced leaf senescence. We found a negative JA regulator, MdBT2, which interacts with MdZAT10 and modulates its stability, thereby repressing MdZAT10-mediated leaf senescence. In summary, we used protein–protein interactions to delineate the relationships among MdBT2, MdZAT10, and MdABI5 during leaf senescence.

## Results

### MdZAT10 positively regulates leaf senescence

The expression of a large number of C2H2-type TFs is markedly induced during natural senescence^19^. The *MdZAT10* gene, a C2H2-type zinc finger TF in subclass C1-2i, is homologous to *Arabidopsis STZ/ZAT10* (*SALT TOLERANCE ZINC FINGER*). The *MdZAT10* (MDP0000198015) gene was identified by a BLAST search against the apple genome database (Apple Gene Function & Gene Family DataBase version 1.0). To identify proteins homologous to ZAT10, a phylogenetic tree containing sequences from 21 different plant species was constructed. All proteins contained two conserved zinc finger domains and an EAR motif, and MdZAT10 was highly homologous to PbZAT10 from *Pyrus bretschneideri* (Supplementary Fig. S1).

**Fig. 1:**
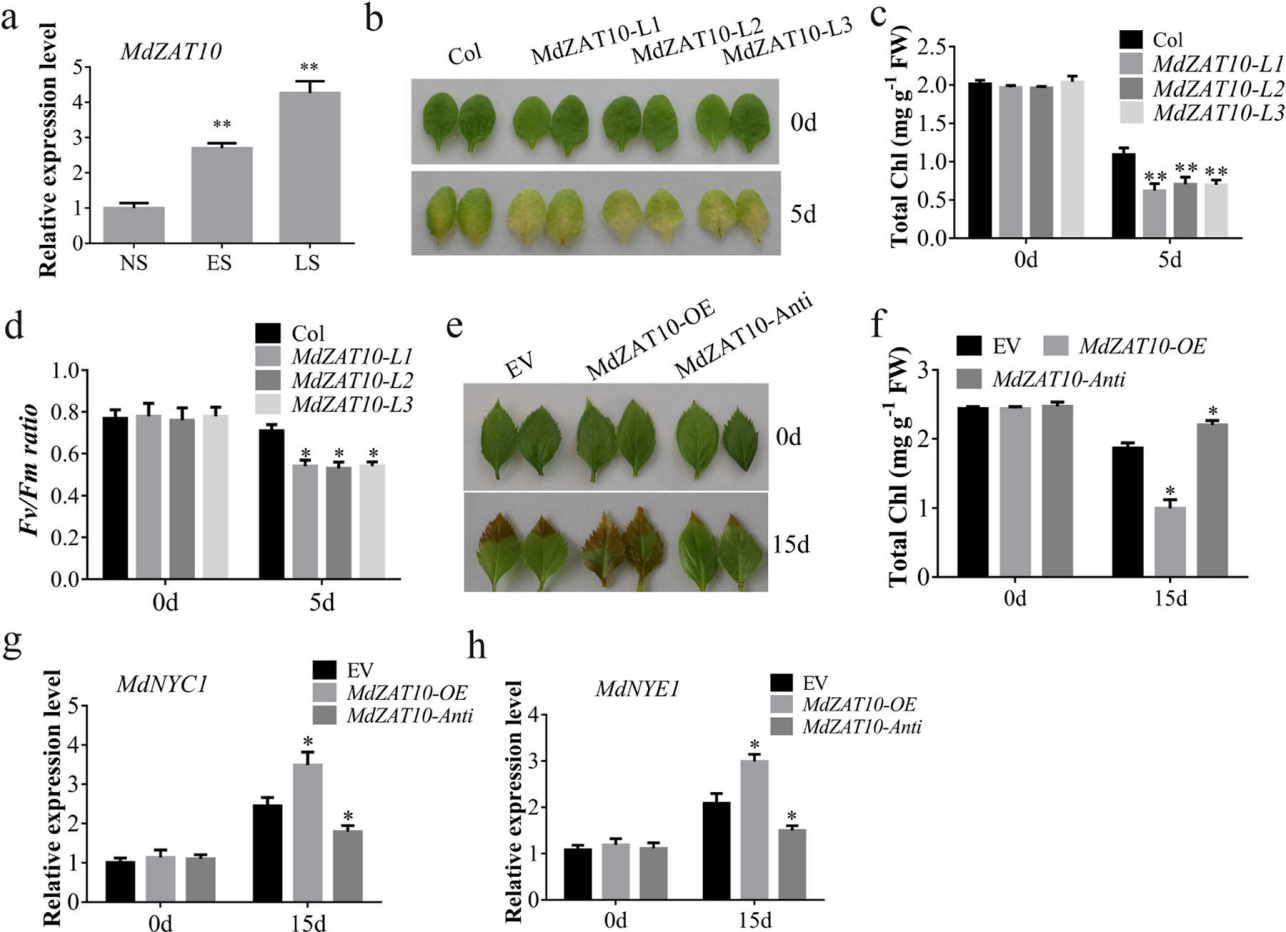
MdZAT10 promotes leaf senescence. **a** MdZAT10 transcript level in late-senescent (LS), early-senescent (ES), and nonsenescent (NS) leaves. **b** Detached leaves from 3-week-old wild-type (Col) plants and three transgenic lines (*MdZAT10*-*L1*, *L2*, and *L3*) were incubated in 3 mM MES buffer in the dark for 5 days. **c** The chlorophyll content and **d** *Fv/Fm* before and after dark treatment were determined. **e** Leaf senescence phenotype and **f** the total chlorophyll content of apple leaves transiently expressing empty vector (EV), overexpressing *MdZAT10* or *MdZAT10* antisense vector in the dark for 15 days. **g**, **h** The expression levels of *MdNYC1* and *MdNYE1* in apple leaves after 15 days of dark treatment. Asterisks indicate significant differences determined by *t*-test (^*^P < 0.05, ^**^P < 0.01)

We measured the expression level of *MdZAT10* in apple leaves at different developmental stages, including the nonsenescent (NS), early-senescent (ES), and late-senescent (LS) stages. *MdZAT10* expression was higher in at ES and LS stages than at the NS stage (Fig. 1a). To confirm the function of MdZAT10 during leaf senescence, an *MdZAT10* overexpression vector was transformed into *Arabidopsis*, generating three independent *Arabidopsis* lines (*MdZAT10*-*L1*, *L2* and *L3*) (Supplementary Fig. S2). Detached leaves from transgenic *Arabidopsis* seedlings showed greater leaf yellowing than those from wild-type (Col) seedlings (Fig. 1b). Consistent with their differences in color, the leaves of the transgenic plants showed significant decreases in chlorophyll content and maximum quantum yield of photosystem II (*Fv/Fm*) (Fig. 1c, d). To further confirm these leaf senescence phenotypes, *MdZAT10* overexpression and antisense suppression vectors were transformed into detached apple leaves using a transient expression system (Supplementary Fig. S2). Consistently, apple leaves overexpressing *MdZAT10* exhibited early senescence, whereas apple leaves expressing *MdZAT10* antisense suppression showed delayed senescence (Fig. 1e). Furthermore, apple leaves’ *MdZAT10* overexpression and antisense suppression vectors also showed corresponding differences in chlorophyll content (Fig. 1f). We found that *MdZAT10* overexpression increased the expression of *MdNYC1* and *MdNYE1* in apple leaves (Fig. 1g, h). These results suggest that MdZAT10 positively regulates leaf senescence.

### MdZAT10 interacts with the MdABI5 protein

**Fig. 2:**
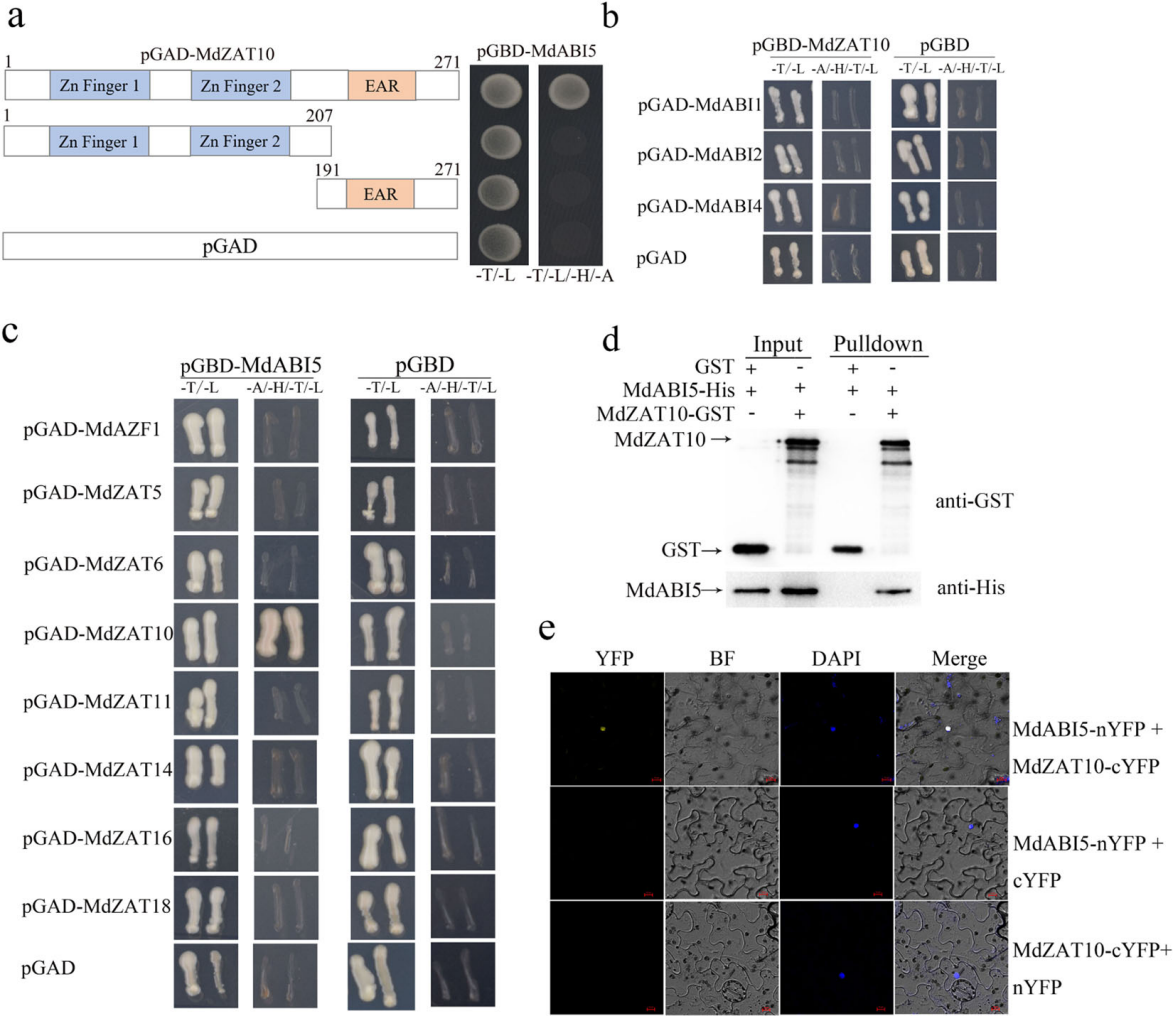
MdZAT10 physically interacts with MdABI5. **a** A yeast two-hybrid (Y2H) assay showed that MdZAT10 interacts with MdABI5. Full-length MdZAT10 and truncated MdZAT10 sequences were cloned into the pGAD424 vector. Full-length MdABI5 was cloned into the pGBT9 vector. The empty pGAD vector was used as a negative control. **b** A Y2H assay showed that MdABIs (MdABI1, MdABI2, and MdABI4) and MdZAT10 interact. **c** A Y2H assay showed that MdABI5 interacts with MdZAT proteins (MdAZF1, MdZAT5, MdZAT6, MdZAT10, MdZAT11, MdZAT14, MdZAT16, and MdZAT18). **d** In vitro MdZAT10 and MdABI5 pull-down assay. The MdABI5-His protein was incubated with MdZAT10-GST and GST. Proteins pulled down with GST beads were detected using anti-GST and anti-His antibodies. **e** BiFC assay. The MdZAT10-cYFP and MdABI5-nYFP constructs were transiently expressed in *Nicotiana benthamiana* leaves, and the fluorescence signal was observed by fluorescence microscopy. Nuclei are indicated by DAPI staining. Scale bars, 10 μm

To further explore the mechanism by which MdZAT10 promotes leaf senescence, a liquid chromatography/mass spectrometry (LC/MS) assay was carried out to screen proteins that interact with MdZAT10 using MdZAT10-GFP as bait. After screening, the MdABI5 protein (GenBank accession number: LOC103430245) was found to interact with MdZAT10, and a yeast two-hybrid (Y2H) assay was performed to confirm this interaction. The full-length cDNA of MdZAT10 was fused to the pGAD424 vector as prey (pGAD-MdZAT10), and the full-length cDNA of MdABI5 was fused to the pGBT9 vector as bait (pGBD-MdABI5). The pGAD-MdZAT10 and pGBD-MdABI5 plasmids were cotransformed into yeast. The results showed an interaction between the MdZAT10 and MdABI5 proteins (Fig. 2a). To identify the regions in MdZAT10 that interact with MdAB15, MdZAT10 was divided into N-terminus (MdZAT10-N) and C-terminus (MdZAT10-C) fragments. These results indicated that the zinc finger domains and EAR motif of MdZAT10 are essential for the interaction between MdZAT10 and MdABI5 (Fig. 2a). MdZAT10 interacted with MdABI5, but not interacted with MdABI1, MdABI2, or MdABI4 (Fig. 2a, b); MdABI5 also specifically interacted with MdZAT10 but not with other MdZATs (Fig. 2c). In addition, we carried out an in vitro pull-down assay and found that MdABI5-His could be pulled down by the MdZAT10-GST fusion protein (Fig. 2d). Finally, in a BiFC assay, a strong yellow fluorescent protein (YFP) fluorescence signal in the nuclei was observed only when MdZAT10-cYFP and MdABI5-nYFP were cotransformed into *Nicotiana benthamiana* leaves (Fig. 2e). These results indicated that MdZAT10 physically interacts with MdABI5.

### MdABI5 promotes leaf senescence

Previous studies have reported that MdABI5 regulates ABA-induced leaf senescence^32^. We detected the expression level of *MdABI5* in apple leaves at different developmental stages. The *MdABI5* expression level was higher at the ES and LS stages than at the NS stage (Supplementary Fig. S3). To elucidate the role of ABI5 during leaf senescence, an *MdABI5* overexpression vector was transformed into *Arabidopsis*, generating three individual transgenic lines (*MdABI5-L1*, *L2*, and *L3*) (Supplementary Fig. S2). *MdABI5* overexpression clearly promoted leaf yellowing and reduced the chlorophyll content and *Fv/Fm* after 5 days in the dark (Supplementary Fig. S3). Furthermore, the *MdABI5* overexpression and *MdABI5* antisense suppression vectors were transiently transformed into detached apple leaves. Apple leaves that overexpressed *MdABI5* showed a more severe senescence phenotype and lower chlorophyll content, whereas *MdABI5* antisense suppression showed a delayed senescence phenotype and higher chlorophyll content (Supplementary Fig. S3). Previous studies have shown that ABI5 induces leaf senescence by directly regulating the expression of the chlorophyll degradation genes *NYC1* and *NYE1*^4,31^. In this study, the expression of *MdNYC1* and *MdNYE1* was enhanced in apple leaves overexpressing *MdABI5* (Supplementary Fig. S3). These results suggest that MdABI5 promotes leaf senescence.

### MdZAT10 promotes MdABI5-regulated leaf senescence

**Fig. 3:**
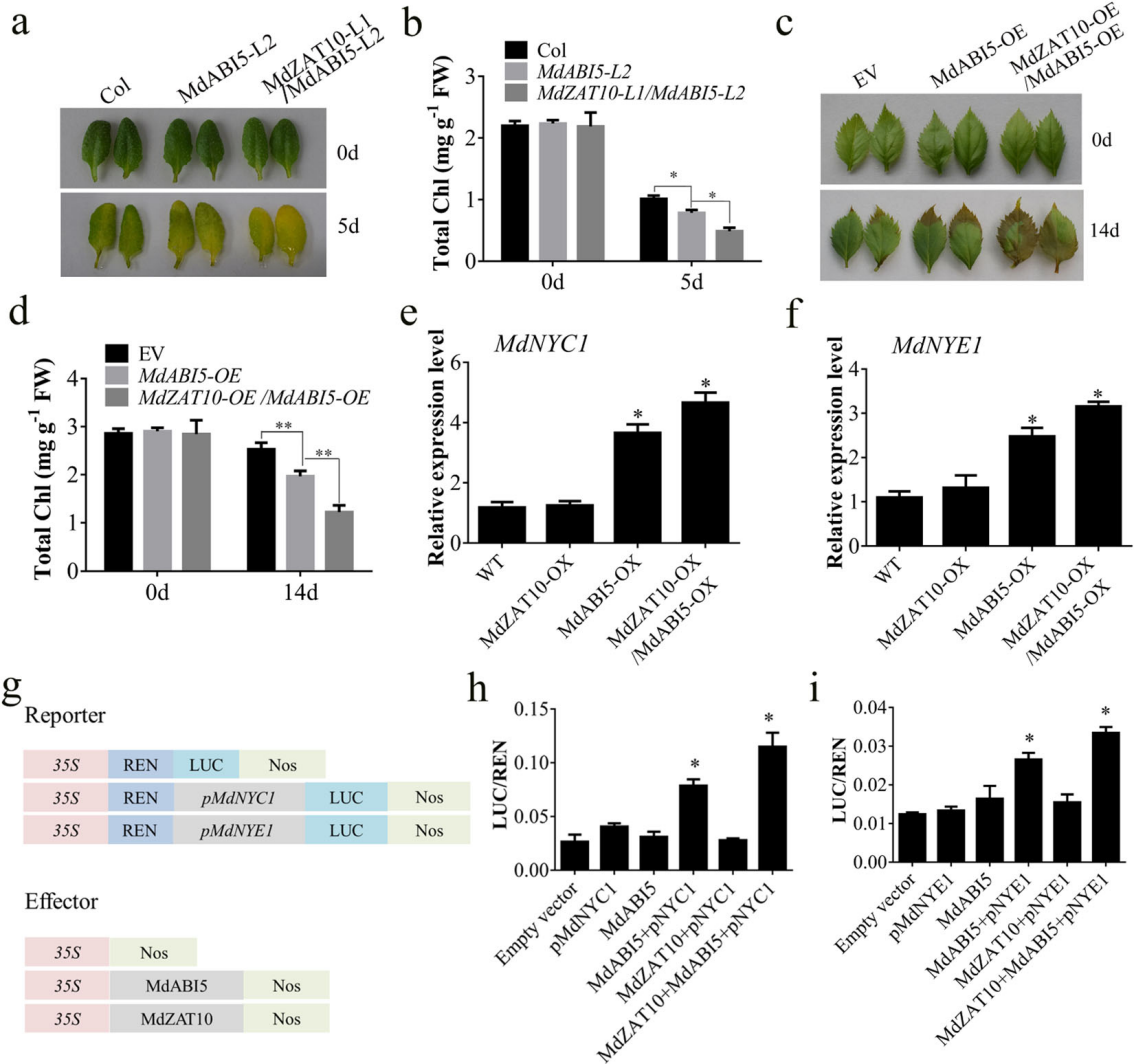
MdZAT10 accelerates MdABI5-regulated leaf senescence. **a** Leaf senescence phenotype and **b** total chlorophyll content of detached leaves from Col, *MdABI5-L2*, and *MdZAT10-L1/MdABI5-L2* transgenic *Arabidopsis* floated on 3 mM MES buffer in the dark for 5 days. **c** Leaf senescence phenotype and **d** total chlorophyll content of apple leaves transiently expressing empty vector (EV), overexpressing *MdABI5* alone or overexpressing *MdZAT10* and *MdABI5* that were floated on 3 mM MES buffer in the dark for 14 days. **e**, **f** The expression levels of *MdNYC1* and *MdNYE1* in wild-type (WT) calli and *MdABI5-OX*, *MdZAT10-OX*, *MdZAT10-OX/MdABI5-OX* transgenic calli were measured by qRT-PCR assay. **g** Schematic diagram of reporter and effector constructs. **h**, **i** A luciferase assay showed that transient cotransformation of MdZAT10 and MdABI5 into tobacco leaves activated the expression of *MdNYC1* and *MdNYE1*. The empty vector (SK + LUC) served as a negative control. The LUC/REN ratio represents the ability of MdABI5 to activate *MdNYC1* and *MdNYE1* expression. Asterisks indicate significant differences at ^*^P < 0.05, ^**^P < 0.01

Given the interaction between MdZAT10 and MdABI5, we suspected that MdZAT10 participates in MdABI5-mediated leaf senescence. An *Arabidopsis* line overexpressing *MdZAT10* was crossed with an *Arabidopsis* line overexpressing *MdABI5*. The resultant *Arabidopsis* plants overexpressing *MdZAT10/MdABI5* turned yellow much faster and had less chlorophyll content than the plants overexpressing *MdABI5* alone (Fig. 3a, b). Consistent with the observed phenotype in *Arabidopsis*, the overexpression of *MdZAT10* increased the MdABI5-mediated leaf senescence in apple leaves (Fig. 3c, d). These results revealed that MdZAT10 accelerates MdABI5-promoted leaf senescence.

Therefore, we hypothesized that MdZAT10 affects the transcriptional activation of *MdNYC1* and *MdNYE1* by MdABI5. To confirm this hypothesis, gene expression in the calli of transgenic apple plants overexpressing *MdABI5* (*MdABI5-OX*) was detected (Supplementary Fig. S2). We found that *MdNYC1* and *MdNYE1* expression was dramatically upregulated in *MdABI5-OX* calli. The *MdZAT10* overexpression vector was introduced into *MdABI5*-overexpressing transgenic calli, and the resultant *MdZAT10-OX/MdABI5-OX* transgenic calli showed markedly increased *MdNYC1* and *MdNYE1* expression (Fig. 3e, f). To confirm this result, we performed a transient expression assay in tobacco leaves. The promoter fragments of *MdNYC1* and *MdNYE1* were fused into the pGreenII 0800-LUC reporter (*pMdNYC1-LUC*, *pMdNYE1-LUC*), and MdZAT10 and MdABI5 were fused into the effector construct pGreenII 62-SK (MdZAT10*-*SK, MdABI5-SK). We found that MdABI5 activated the promoters of *MdNYC1* and *MdNYE1* and had a stronger effect when MdZAT10 and MdABI5 were cotransformed (Fig. 3g–i). These results indicated that the interaction between MdZAT10 and MdABI5 enhances the transcriptional activity of MdABI5 for *MdNYC1* and *MdNYE1*.

### MdZAT10 activates JA-induced leaf senescence

**Fig. 4:**
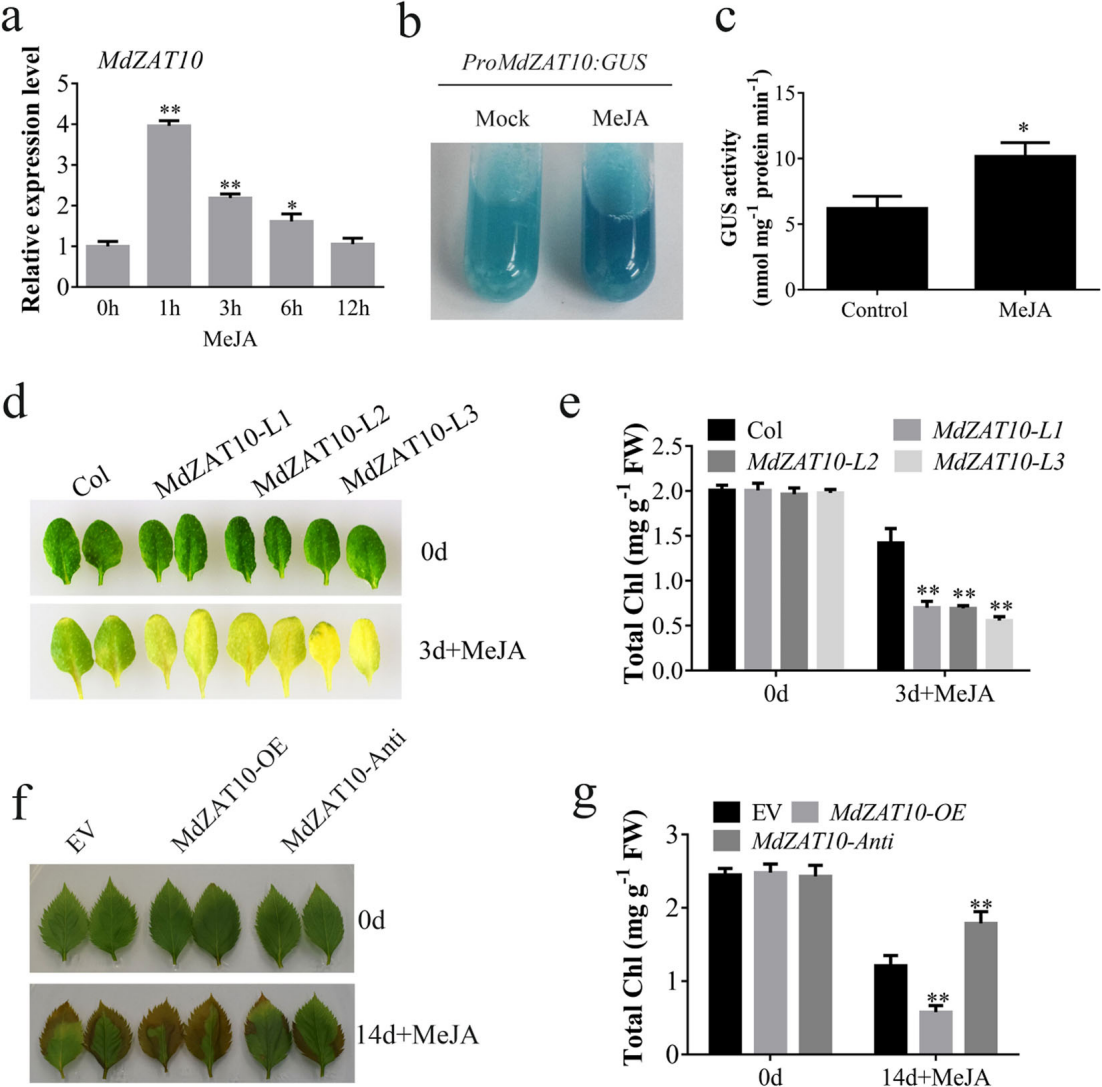
MdZAT10 accelerates JA-induced leaf senescence. **a** Transcript level of *MdZAT10* in response to 100 μM MeJA. **b** GUS staining and **c** GUS activity in *proMdZAT10::GUS* transgenic calli with (MeJA) or without (Mock) 100 µM MeJA treatment for 12 h. **d** Leaf senescence phenotype and **e** chlorophyll content of detached leaves from 3-week-old Col and transgenic *Arabidopsis MdZAT10-L1*, *L2*, and *L3* floated on 3 mM MES buffer with or without 100 μM MeJA for 3 days. **f** Leaf senescence phenotype and **g** chlorophyll content of apple leaves transiently expressing empty vector (EV), overexpressing *MdZAT10* or *MdZAT10* antisense vector floated on 3 mM MES buffer with or without 100 μM MeJA for 14 days. Asterisks indicate significant differences by *t*-test (^*^P < 0.05, ^**^P < 0.01)

We also found that *MdZAT10* was induced by MeJA (Fig. 4a). To further confirm the function of *MdZAT10* in response to JA, we produced transgenic apple calli that expressed *β-glucuronidase* (*GUS*) driven by a region 2 kb upstream of the *MdZAT10* gene. Histochemical staining showed that MeJA treatment significantly increased GUS activity (Fig. 4b, c). Detached leaves from *MdZAT10* transgenic *Arabidopsis* showed increased leaf yellowing with MeJA treatment (Fig. 4d). The chlorophyll content in these leaves was significantly reduced compared to the leaves of Col plants (Fig. 4e). Consistently, upon MeJA treatment, apple leaves overexpressing *MdZAT10* also exhibited early senescence, whereas *MdZAT10* antisense suppression showed delayed senescence (Fig. 4f). Furthermore, leaves *MdZAT10* overexpression and *MdZAT10* antisense suppression showed different chlorophyll contents (Fig. 4g). These results indicated that MdZAT10 acts as a positive regulator of JA-induced leaf senescence in apple.

### MdBT2 physically interacts with the MdZAT10 protein

In addition to its transcriptional regulation, we found that MdZAT10 was regulated at the posttranslational level in response to MeJA treatment. An in vitro protein degradation assay was performed to measure the protein level of MdZAT10 in response to MeJA treatment. The fusion protein MdZAT10-His was incubated with total protein from apple calli with or without MeJA treatment. The MdZAT10-His level dropped rapidly without MeJA treatment, but the drop in the MdZAT10 protein level was markedly abrogated by treatment with MeJA or the 26S proteasome inhibitor MG132 (Supplementary Fig. S4a). Furthermore, the MdZAT10 protein level in *MdZAT10*-overexpressing transgenic apple calli increased with MeJA treatment (Supplementary Fig. S4b). These results indicated that the presence of MeJA reduced MdZAT10 protein degradation by the 26S proteasome pathway.

**Fig. 5:**
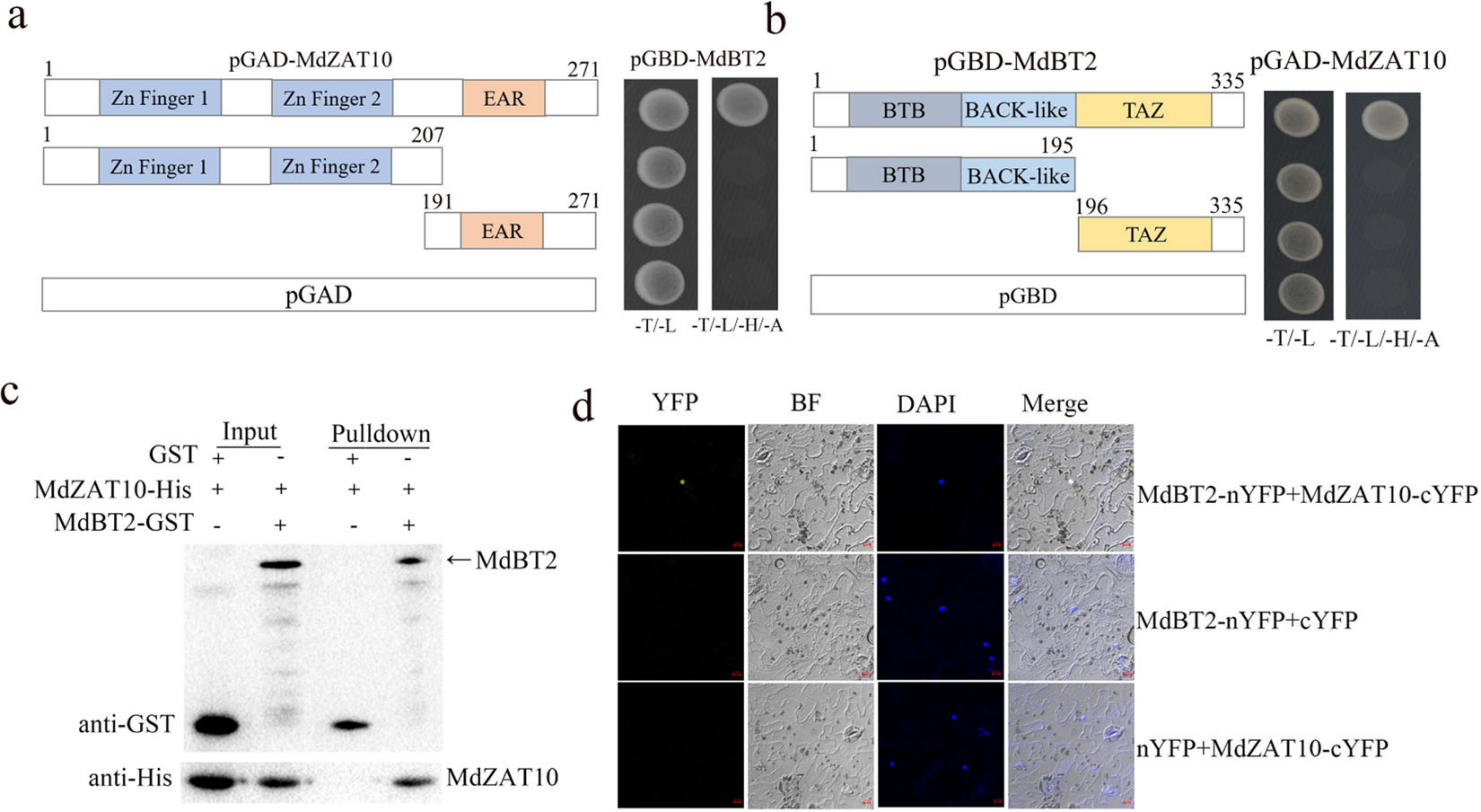
MdZAT10 physically interacts with MdBT2. **a**, **b** A Y2H assay showed the interaction between MdZAT10 and MdBT2. Full-length and truncated MdZAT10 and MdBT2 sequences were cloned into the pGBD and pGAD vectors. **c** In vitro pull-down assay with MdZAT10 and MdBT2. The MdZAT10-His protein was incubated with GST-MdBT2 and GST. Proteins that were pulled down with GST beads were detected using anti-GST and anti-His antibodies. **d** BiFC assay. The MdZAT10-cYFP and MdBT2-nYFP constructs were transiently expressed in *Nicotiana benthamiana* leaves, and the fluorescence signal in the nucleus was observed via fluorescence microscopy. Nuclei are indicated by DAPI staining. Scale bars, 10 μm

In addition to MdABI5, MdBT2 was screened as a potential interaction protein of MdZAT10. MdBT2 plays a key role in the regulation of JA-mediated leaf senescence^36^. We used a Y2H assay to determine whether MdBT2 and MdZAT10 interact. The full-length cDNA of MdBT2 was fused to the pGBT9 vector as bait (pGBD-MdBT2). Only yeast cells that contained pGAD-MdZAT10 and pGBD-MdBT2 grew well on -Trp/-Leu/-His/-Ade screening medium (Fig. 5a). To determine the regions of MdBT2 that interact with MdZAT10, MdBT2 was divided into N-terminal (MdBT2-N) and C-terminal (MdBT2-C) fragments. As shown in Fig. 5b, the BTB and TAZ domains of MdBT2 were indispensable for this interaction (Fig. 5b). To determine whether MdBT2 specifically interacts with MdZAT10, seven apple C2H2-type ZFPs (MdAZF1, MdZAT5, MdZAT6, MdZAT11, MdZAT14, MdZAT16, and MdZAT18) were fused to the prey vector pGAD424. However, all of them failed to interact with MdBT2 (Supplementary Fig. S5). MdBT1, MdBT2, MdBT3.1, and MdBT4 are important members of the apple MdBT protein family, and MdBT1 and MdBT2 interact with MdZAT10 (Supplementary Fig. S5).

The MdBT2-MdZAT10 interaction was further verified by pull-down and BiFC assays. For the pull-down assay, the fusion protein MdBT2-GST and GST as a control were incubated with the fusion protein MdZAT10-His. Only MdBT2-GST could pull down MdZAT10-His (Fig. 5c). Next, we conducted a BiFC assay to further confirm the interaction. MdZAT10 and MdBT2 were fused to the C-terminal (cYFP) and N-terminal (nYFP) regions of yellow fluorescent protein, respectively. As shown in Fig. 5d, a strong fluorescence signal was detected in the nucleus when MdZAT10-cYFP and MdBT2-nYFP were injected into *N.* *benthamiana* leaves, whereas no YFP fluorescence signal was detected in the negative controls. Taken together, these results suggested that MdBT2 physically interacts with MdZAT10.

### MdBT2 promotes degradation of the MdZAT10 protein

Recent studies have shown that MdBT2 generally negatively regulates the stability of its target proteins by ubiquitination^37,38^. We incubated the MdZAT10-His fusion protein with total protein from apple calli treated with or without the proteasome inhibitor MG132. The protein stability of MdZAT10 was enhanced by MG132, indicating that the MdZAT10 protein is degraded by the 26S proteasome pathway (Supplementary Fig. S4).

**Fig. 6:**
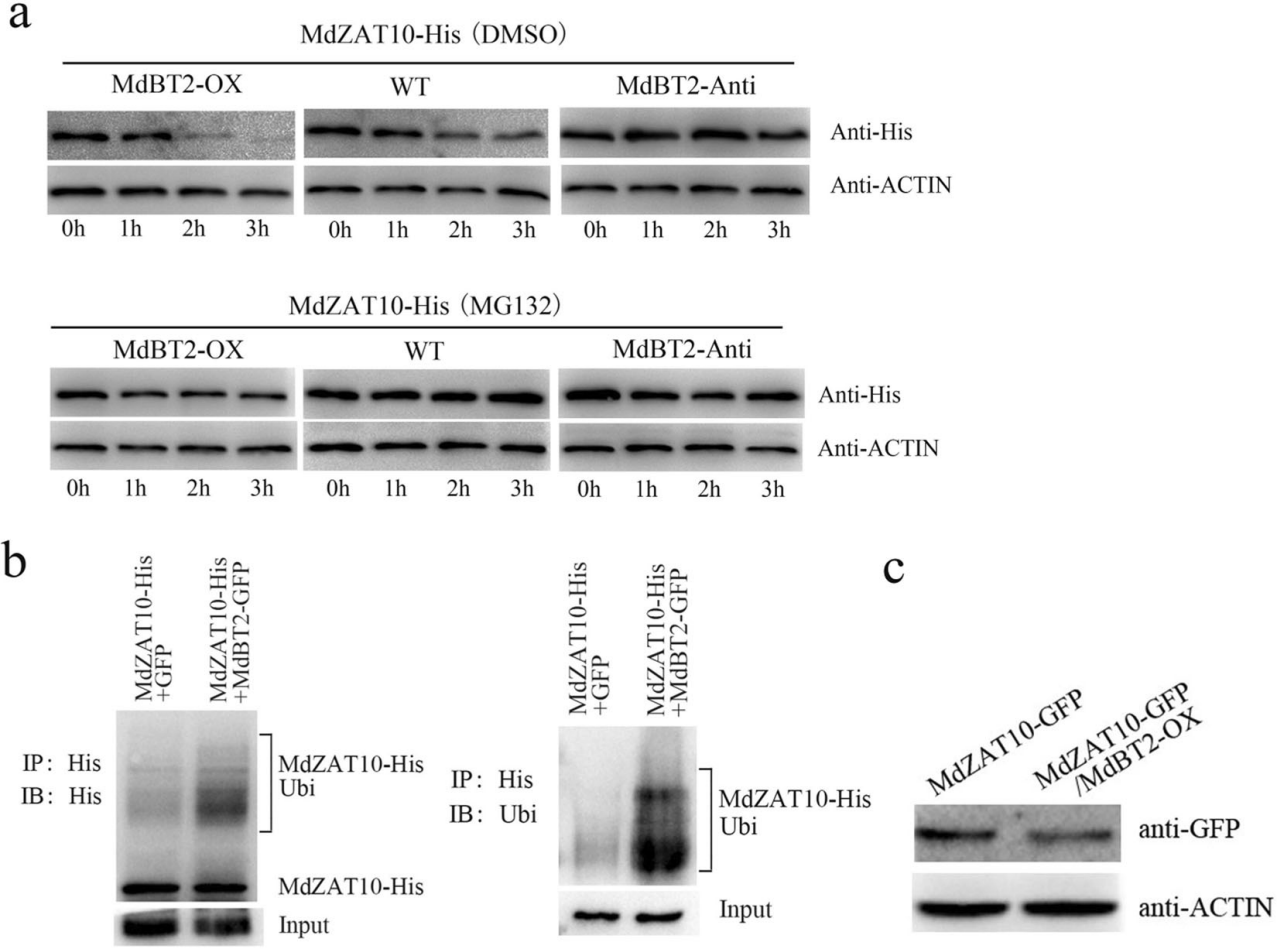
MdBT2 mediates MdZAT10 protein stability. **a** Cell-free MdZAT10-His degradation assay. The MdZAT10-His fusion protein was incubated with total protein extracted from wild-type (WT), *MdBT2-OX*, and *MdBT2-Anti* transgenic calli for the indicated time periods. **b** MdBT2 ubiquitinated the MdZAT10-His protein in vivo. MdZAT10-His was immunoprecipitated using an anti-His antibody and then examined using anti-His and anti-Ubi antibodies. IP, immunoprecipitate; IB, immunoblot; Ubi, ubiquitin. **c** The MdZAT10-GFP protein abundance in transgenic apple calli (*MdZAT10-GFP* and *MdZAT10-GFP/MdBT2-OX*) was assessed by immunoblotting using an anti-GFP antibody

Then, we speculated that MdBT2 mediates MdZAT10 protein stability. To confirm this speculation, we generated transgenic *MdBT2*-overexpressing and antisense apple calli (*MdBT2-OX* and *MdBT2*-*Anti*). A cell-free MdZAT10 degradation assay was performed by incubating MdZAT10-His with total protein extracted from wild-type, *MdBT2-OX*, and *MdBT2*-*Anti* calli. As shown in Fig. 6a, degradation of the MdZAT10-His protein was much more rapid in the *MdBT*2*-OX* calli than in the wild-type calli, whereas MdZAT10-His degraded more slowly in the *MdBT2-Anti* calli. However, the proteasome inhibitor MG132 noticeably repressed degradation of the MdZAT10-His protein (Fig. 6a). These results indicated that the MdZAT10 protein is degraded by the 26S proteasome pathway, and a ubiquitination assay was performed to further verify this finding. The total protein extracted from *MdBT2-GFP* calli and GFP was incubated with the MdZAT10-His protein. The ubiquitinated MdZAT10-His protein was assessed using anti-His and anti-Ubi antibodies. A larger amount of high-molecular-mass MdZAT10-His was detected in the *MdBT2-GFP* + MdZAT10-His mixture (Fig. 6b). To confirm that MdBT2 promotes the degradation of MdZAT10 in vivo, we generated *35S::MdZAT10-GFP* and *35S::MdZAT10-GFP* + *35S::MdBT2-OX* transgenic apple calli. Western blotting was performed with an anti-GFP antibody, and the *MdZAT10*-*GFP* protein abundance was lower in the *35S::MdZAT10-GFP* + *35S::MdBT2-OX* transgenic calli (Fig. 6c). Taken together, these results demonstrated that MdBT2 mediates degradation of the MdZAT10 protein.

### MdBT2 delayed the MdZAT10-mediated leaf senescence

**Fig. 7:**
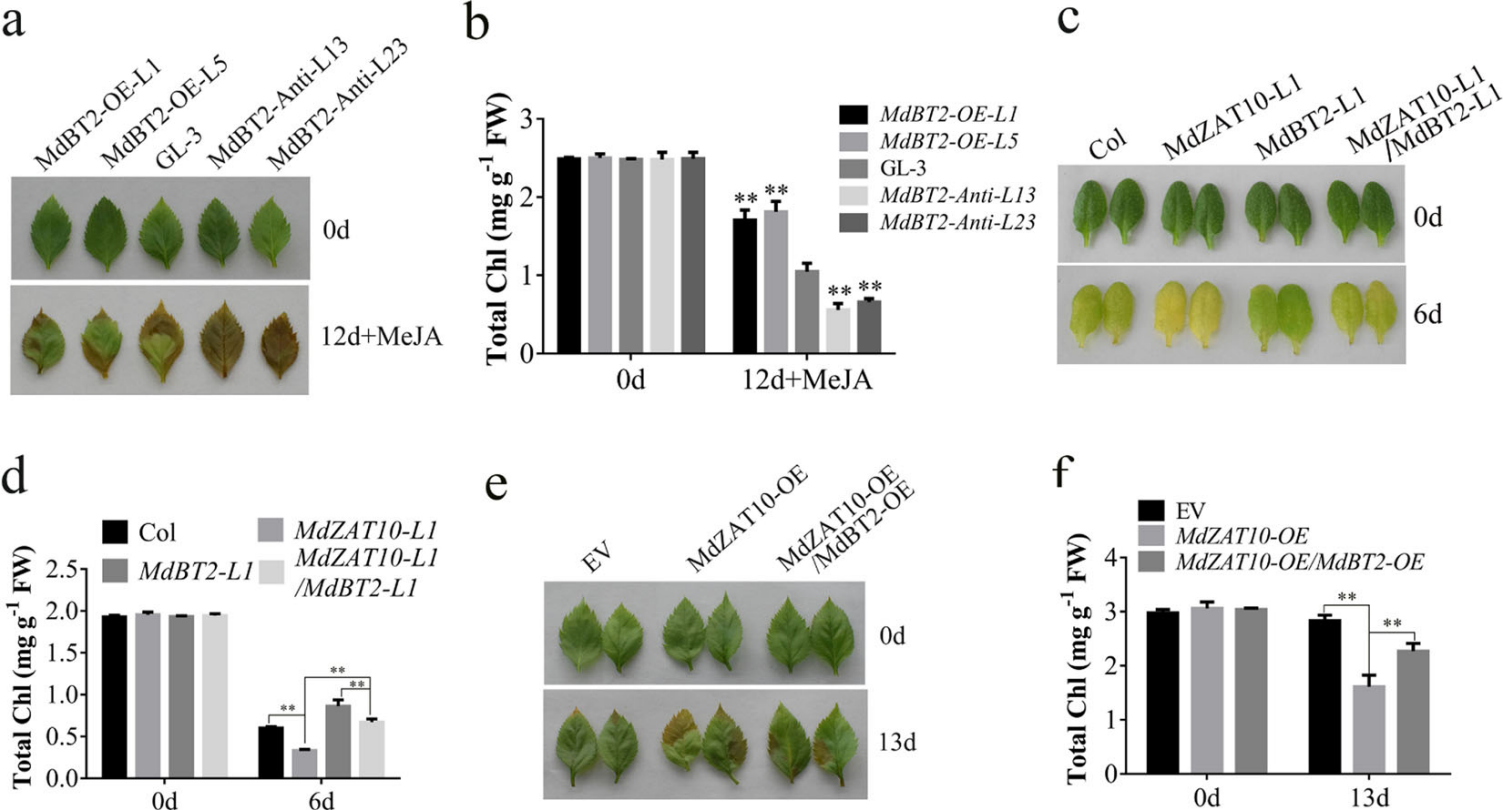
MdBT2 delayed the MdZAT10-promoted leaf senescence. **a** Leaf senescence phenotype and **b** chlorophyll content of detached leaves from wild-type (GL-3) and transgenic apple seedlings overexpressing *MdBT2* (*MdBT2-OE-L1*, *MdBT2-OE-L5*) and *MdBT2* antisense (*MdBT2-Anti-L13*, *MdBT2-Anti-L23*) that were floated on 3 mM MES buffer with 100 μM MeJA in the dark for 12 days. **c** Leaf senescence phenotype and **d** chlorophyll content of detached leaves from Col and transgenic *Arabidopsis MdZAT10-L1*, *MdZAT10-L1/MdBT2-L1*, and *MdZAT10-L1/MdBT2-L2* that were floated on 3 mM MES buffer in the dark for 6 days. **e** Leaf senescence phenotype and **f** chlorophyll content of apple leaves transiently expressing empty vector (EV) or overexpressing *MdZAT10* alone or *MdZAT10* and *MdBT2* were floated on 3 mM MES buffer in the dark for 13 days. Asterisks indicate significant differences at ^*^P < 0.05, ^**^P < 0.01

Previous studies have revealed that MdBT2 delays JA-induced leaf senescence^36^. Given that MdBT2 promoted the ubiquitination of MdZAT10, we speculated that MdBT2 is involved in the regulation of MdZAT10-promoted leaf senescence. To investigate the potential function of MdBT2 in leaf senescence, we obtained three transgenic *Arabidopsis* lines (*MdBT2*-*L1*, *L2*, and *L3*) and transgenic apple plants overexpressing *BT2* (*MdBT2-OE-L1* and *MdBT2-OE-L5*) and *BT2* antisense (*MdBT2-Anti-L13* and *MdBT2-Anti-L23*) (Supplementary Fig. S2). *MdBT2*-overexpressing leaves from both *Arabidopsis* and apple showed a delayed senescence phenotype, whereas MdBT2 antisense plants showed accelerated JA-induced leaf senescence. The chlorophyll content was consistent with the phenotype (Fig. 7a, b and Supplementary Fig. S6). The *Arabidopsis* line overexpressing *MdZAT10* was crossed with the *Arabidopsis* line overexpressing *MdBT2*. The resultant *Arabidopsis* plants overexpressing *MdZAT10/MdBT2* showed delayed leaf senescence and enhanced chlorophyll content compared to those of plants overexpressing *MdZAT10* (Fig. 7c, d). Consistent with these results, the overexpression of *MdBT2* decreased the MdZAT10-promoted leaf senescence phenotype in apple leaves and increased the chlorophyll content (Fig. 7e, f). Taken together, these results indicated that MdBT2 delayed the MdZAT10-promoted leaf senescence.

## Discussion

Leaf senescence is a complex process that involves the degradation of cellular components such as chloroplasts^39^. Accompanied by the degradation of massive amounts of chlorophyll, the most visible feature of plant senescence is leaf yellowing. Leaf senescence also affects crop productivity and plant fitness^40^. Many TFs show expression changes during leaf senescence^41^. In this study, we identified a C2H2-type zinc finger TF, MdZAT10, which positively regulates dark- and JA-induced leaf senescence.

STZ/ZAT10 is a member of the C2H2-type zinc finger TF family in subclass C1-2i that is involved in different abiotic stresses such as drought, salinity, cold, and osmotic stresses^42–46^. In addition, it plays central roles in plant growth and development^47^. Previous studies showed that several C2H2-type zinc finger TFs are induced during senescence in *Arabidopsis*^19,41^. AZF2 functions as a positive regulator of age-dependent leaf senescence, and the loss of AZF2 function delayed leaf senescence^27^. Our results showed that *MdZAT10* expression is higher in senescent leaves than in young leaves (Fig. 1), and overexpressing *MdZAT10* accelerated leaf senescence (Fig. 1). We further explored how MdZAT10 promotes leaf senescence and measured the expression levels of some senescence-related genes. *MdZAT10-OX* transgenic calli showed significantly enhanced the expression of senescence-related genes *MdSAG29* and *MdPAO* and downregulated the expression of *MdWRKY70* and *MdAPX2* (Supplementary Fig. S7). Overexpression of *SAG29* in *Arabidopsis* accelerated leaf senescence^48^. *SAG29* acts as a target gene of MYC2 and is involved in JA-induced leaf senescence^18^. *Pheophorbide a oxygenase* (*PAO*) is induced by natural senescence, and the *pao1* mutant exhibits a stay-green phenotype^49^. In *Arabidopsis*, WRKY70 negatively regulates age-dependent leaf senescence^50^. *APX2* (*ASCORBATE PEROXIDASE2*) encodes cytosolic APX2, which plays a key role in removing H_2_O_2_^51^. H_2_O_2_ is the most commonly used inducer of leaf senescence^52^. Elevated reactive oxygen species (ROS) levels have been reported to accelerate leaf senescence^53–55^. It is possible that MdZAT10 promotes leaf senescence by reducing ROS scavenging or regulating the expression of senescence-related genes.

In addition, we found that MdZAT10 promotes JA-induced leaf senescence. MdZAT10 was induced with MeJA treatment, and *MdZAT10-OX* transgenic calli showed an enhanced expression of *MdAOC1* and *MdAOS* (*ALLENE OXIDE SYNTHASE*) (Supplementary Fig. S7). AOC1 and AOS are associated with the JA biosynthesis pathway, and their expression is upregulated during leaf senescence^10^. In *Arabidopsis*, MYC2 binds the promoter of *STZ/ZAT10* and regulates its expression^56^. STZ/ZAT10 and AZF2 can bind the *LOX3* promoter to regulate the early response to MeJA^57^. These results indicate that STZ/ZAT10 is involved in JA signaling.

In addition to regulating downstream genes, MdZAT10 may regulate leaf senescence by interacting with other proteins. Here, we demonstrated that MdZAT10 interacts with MdABI5 (Fig. 2). ABI5 regulates dark- and ABA-induced leaf senescence^4,30,32,58^. Recent studies have indicated that ABI5 negatively regulates photosynthesis and chloroplast development under dark treatment^4,58^. RNA-seq data showed that StABI5 negatively regulates the expression level of photosynthesis-related genes^58^. ABI5 positively regulates leaf senescence by directly repressing the expression of *ABR*^30^ and activating the expression of *NYC1* and *NYE1*^4,31,58^. Here, our data showed that *MdABI5*-overexpressing transgenic plants showed enhanced leaf senescence and that *MdABI5* antisense suppression delayed leaf senescence (Supplementary Fig. S3). MdABI5 activated the expression of *MdNYC1* and *MdNYE1* in *MdABI5-OX* transgenic apple calli, further confirming the positive regulation of leaf senescence by MdAB15 (Fig. 3). We found that MdZAT10 accelerated MdABI5*-*mediated leaf senescence and increased the transcriptional activation of *MdNYC1* and *MdNYE1* by MdABI5 (Fig. 3). It is possible that MdZAT10 promotes leaf senescence by interacting with MdABI5 to affect the transcriptional activity of MdABI5 for target genes.

In addition to MdABI5, MdZAT10 also interacts with MdBT2. MdBT2 delayed JA-induced leaf senescence, and MdBT2 delayed the MdZAT10-promoted leaf senescence (Fig. 7). These results indicated that MdBT2 plays an opposite role in MdZAT10-promoted leaf senescence. Ubiquitination is widely involved in plant biological processes^20^. Several studies have shown the involvement of some E3 ligases in leaf senescence via the 26S proteasome pathway^59^. A recent study revealed that MdBT2 interacts with MdMYC2 and MdJAZ2, thereby regulating JA-induced leaf senescence^36^. BT2 is a component of the CRL3 complex that promotes target protein ubiquitination^60^. Our results showed that MdBT2 promoted the degradation of MdZAT10 and delayed JA-induced leaf senescence (Figs. 5–7). In the presence of JA, the degradation of MdBT2 was promoted^36^, thus releasing MdZAT10 from MdBT2-mediated degradation. Our results provide insight into the molecular mechanisms by which BT2 mediates JA-induced leaf senescence. These results imply that MdBT2 dynamically regulates JA-induced leaf senescence by regulating different target proteins. Although MdZAT10 interacts with MdBT2 and MdABI5, it is unclear whether these two interactions are related. The same region of MdZAT10 interacts with MdBT2 and MdABI5, and both MdBT2 and MdABI5 interact with full-length MdZAT10 (Figs. 2 and 5). It is possible that MdBT2 and MdABI5 competitively interact with MdZAT10 to antagonistically regulate leaf senescence, but this hypothesis requires further verification.

**Fig. 8:**
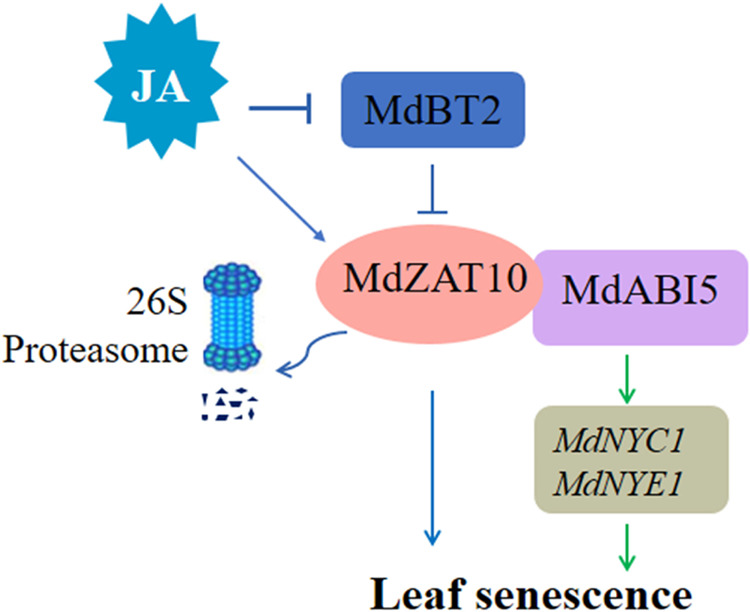
A working model of the role of MdZAT10 in apple leaf senescence. On the one hand, MdZAT10 positively regulates leaf senescence by interacting with MdABI5, enhancing the transcriptional activity of MdABI5 for MdNYC1 and MdNYE1. On the other hand, in the absence of JA, MdBT2 interacts with MdZAT10 and ubiquitinates MdZAT10 to degrade MdZAT10, thereby negatively regulating MdZAT10-promoted leaf senescence. In the present of JA, JA accelerates the degradation of MdBT2, releasing MdZAT10, which contributes to JA-induced leaf senescence.

In summary, a working model to summarize the role of MdZAT10 in leaf senescence is proposed (Fig. 8). On the one hand, MdZAT10 positively regulates leaf senescence through its interaction with MdABI5, enhancing the transcriptional activity of MdABI5 for the chlorophyll degradation genes *MdNYC1* and *MdNYE1*. On the other hand, in the absence of MeJA, MdBT2 interacts with MdZAT10 and ubiquitinates MdZAT10 to degrade MdZAT10, thereby negatively regulating MdZAT10-promoted leaf senescence. In contrast, *MdZAT10* is induced by exogenous MeJA, which promotes leaf senescence. MeJA accelerates the degradation of MdBT2, releasing MdZAT10, which contributes to JA-induced leaf senescence. In this study, we have characterized the role of MdZAT10 in the leaf senescence regulatory network through its direct interaction with MdBT2 and MdABI5.

## Materials and methods

### Plant materials and growth conditions

Tissue cultures of apple (*Malus* × *domestica* ‘GL-3’) were used in this study. The apple seedlings were subcultured on MS medium supplemented with 0.6 mg L^−1^ 6-BA, 0.2 mg L^−1^ GA, and 0.2 mg L^−1^ NAA under long-day conditions (25 °C, 16/8 h light/dark) and subcultured at 30-day intervals. ‘Orin’ apple calli (*Malus domestica* Borkh.) were also used in this study. The calli were cultured on MS medium supplemented with 1.5 mg L^−1^ 2,4-D and 0.4 mg L^−1^ 6-BA at room temperature under dark conditions and subcultured at 15-day intervals. The apple calli were used for genetic transformation. *Arabidopsis thaliana* ecotype Columbia seedlings were grown at 22 °C under long-day conditions (16/8 h light/dark). *Arabidopsis* seedlings were used for genetic transformation and functional identification.

### Vector construction and plant transformation

To construct overexpression vectors, the full-length coding sequences of *MdBT2* and *MdABI5* were cloned into the pCXCN-Myc vector, and the full-length coding sequence *MdZAT10* was cloned into the pRI-101 vector (with a GFP tag). To generate antisense suppression vectors (*MdBT2*-*Anti*, *MdABI5-Anti*, and *MdZAT10*-*Anti*), fragments of the *MdBT2*, *MdABI5*, and *MdZAT10* sequences were cloned into the pRI-101 vector. MdBT2-GFP transgenic apple calli were obtained as described in our previous study^38^. To generate the *proMdZAT10:GUS* construct, the promoter fragment of *MdZAT10* was inserted into the pCAMBIA1300-GUS vector, which was then transformed into apple calli. Transgenic apple seedlings, apple calli, and *Arabidopsis* seedlings were obtained according to previously described methods^61,62^. The transient transformation apple leaves were obtained according to a previously described method^62^. All the primers used for gene cloning are listed in Supplementary Table 1.

### RNA extraction and gene expression analysis

Total RNA was isolated from apple seedlings, apple calli, *Arabidopsis* seedlings, and treated seedlings using the RNAplant Plus kit (TIANGEN) according to the manufacturer’s protocol. First-strand cDNA was synthesized using the PrimeScript™ RT Reagent Kit (TaKaRa) according to the manufacturer’s instructions. qRT-PCR was performed on a StepOnePlus instrument (Applied Biosystems) using UltraSYBR Mixture (Takara). All primers are listed in Supplementary Table 2. Three biological replicates and three technical replicates were performed for each experiment.

### MdZAT10-interacting protein screen

A LC/MS assay was performed with the MdZAT10-GFP protein to screen out MdZAT10-interacting proteins as previously described^63^. The MdZAT10-GFP protein was extracted from *MdZAT10*-overexpressing transgenic calli and purified using a Pierce Classic IP Kit (Thermo Fisher). The MdZAT10-GFP protein was incubated with total protein extracted from apple seedlings for 6 h. The mixed protein solutions were incubated with protein A/G agarose beads and anti-GFP antibody according to a standard co-immunoprecipitation protocol. Then, the resin was eluted, and the eluted proteins were separated on SDS-PAGE gels. Protein identification was carried out by LC-MS/MS (OE Biotech, Shanghai, China).

### Yeast two-hybrid assay

To confirm the interactions between MdZAT10 and MdABI5, and MdBT2 and MdZAT10, the coding sequences of *MdZAT10*, *MdABI5*, and *MdBT2* were cloned into the pGAD424 and pGBT9 vectors to form pGAD-MdZAT10, pGBD-MdABI5, and pGBD-MdBT2. Truncated MdZAT10 sequences (amino acids 1–207 and 191–270) were cloned into pGAD424. The truncated MdBT2 sequences were also cloned into pGBT9^37^. We performed a Y2H assay as described previously^64^. The pGBD-MdABI5 and pGBD-MdBT2 plasmids were individually transformed with pGAD-MdZAT10 into yeast strain Y2H Gold (Clontech). Yeast transformants were grown on SD base/-Leu/-Trp medium and then transferred onto SD base/-Leu/-Trp/-His/-Ade medium for interactions.

### Pull-down and BiFC assays

The full-length coding sequences of *MdZAT10*, *MdBT2*, and *MdABI5* were cloned into the pET32a and pGEX4T-1 vectors to generate the recombinant constructs MdZAT10-pET32a, MdZAT10-pGEX 4T-1, MdABI5-pET32a, and MdBT2-pGEX 4T-1. The constructs were introduced into *Escherichia coli* BL21 (DE3), after which the MdZAT10-His, MdZAT10-GST, MdABI5-His, and MdBT2-GST fusion proteins were generated by induction with 1 mM isopropyl β-D-1-thiogalactopyranoside (IPTG). The eluted proteins were detected by western blotting with anti-GST and anti-His antibodies (Abmart, Shanghai, China).

The coding sequences of *MdZAT10*, *MdBT2*, and *MdABI5* were cloned into the 35S::pSPYCE-cYFP and 35S::pSPYNE-nYFP vectors to generate MdZAT10-cYFP, MdBT2-nYFP, and MdABI5-nYFP. The recombinant constructs were transformed into *Agrobacterium tumefaciens LBA4404* and then injected into *N.* *benthamiana* leaves. YFP fluorescence signals were detected using a confocal laser-scanning microscope (Zeiss).

### Analysis of leaf senescence phenotype

To examine the leaf senescence phenotype, detached leaves were placed on 3 mM MES buffer (pH 5.8) in the dark at 22 °C. To examine phytohormone-induced leaf senescence, detached leaves were floated on 3 mM MES buffer (pH 5.8) containing 100 μM MeJA at 22 °C and kept under dim light for the indicated time period.

### Quantification of the chlorophyll content and *Fv/Fm* ratio

To measure the total chlorophyll concentration, total pigments were extracted from plant leaves with 95% (v/v) ethanol for 24 h. The absorbance at 649 and 665 nm was measured using an ultraviolet/visible spectrophotometer (SOPTOP UV2800S, Shanghai, China). To calculate the *Fv/Fm* ratios, leaves were analyzed with a closed chlorophyll fluorescence imaging system (Photon System Instruments, Brno, Czech Republic) according to the manufacturer’s instructions as previously described^31^.

### Protein degradation and ubiquitination assays

For the in vitro protein degradation assay, total protein was extracted from wild-type and transgenic apple calli using degradation buffer (25 mM Tris-HCl, pH 7.5, 10 mM NaCl, 10 mM MgCl_2_, 4 mM phenylmethylsulfonyl fluoride, 5 mM DTT, and 10 mM ATP). The protein extracts were incubated with MdZAT10-His at 22 °C and assessed by western blotting with an anti-His antibody (Abmart).

We performed an in vivo ubiquitination assay as described previously^64^. In brief, MdBT2-GFP transgenic calli was extracted using a Pierce^TM^ Classic IP Kit (Thermo Fisher), and the extracts were incubated with MdZAT10-His protein at 4 °C overnight. The in vivo ubiquitination of MdZAT10 was detected by western blotting with anti-His (Abmart) and anti-Ubi (Sigma-Aldrich) antibodies.

### Transient expression assay

To carry out the transient expression assay, the *MdNYC1* and *MdNYE1* promoter sequences were inserted into the pGreenII 0800-LUC vector. Full-length *MdZAT10* and MdABI5 were cloned into the pGreen 62-SK vector. The recombinant plasmids were transformed into *N.* *benthamiana* leaves by Agrobacterium-mediated transformation, and LUC/REN activity ratio was detected using a dual-luciferase reporter assay system (Promega)^64^.

### GUS analysis

For GUS staining, transgenic plants were incubated in X-gluc buffer (1 mM X-Gluc, 0.5 mM ferricyanide, 0.5 mM ferrocyanide, 0.1 mM EDTA, and 0.1% Triton X-100) at 37 °C for 12 h. GUS activity was detected using a fluorescence spectrophotometer.

### Statistical analyses

Each experiment in this study was repeated at least three times. The data were analyzed by *t*-test using GraphPad Prism 6.02 software, and asterisks denote significant differences (^*^P < 0.05, ^**^P < 0.01).

### Accession numbers

Sequence data from this article can be found in the Apple Genome (GDR): MdABI5 (LOC103430245), MdABI1 (MDP0000265371), MdABI2 (MD15G1054500), MdABI4 (MD01G1155400), MdBT1 (MDP0000151000), MdBT2 (MDP0000643281), MdBT3.1 (MDP0000296225), MdBT4 (MDP0000215415), MdNYE1, (MDP0000322543), MdNYC1 (MDP0000124013), MdAZF1 (MDP0000265345), MdZAT5 (MDP0000769354), MdZAT6 (MDP0000319225), MdZAT10 (MDP0000198015), MdZAT11 (MDP0000305944), MdZAT14 (MDP0000204390), MdZAT16 (MDP0000137826), and MdZAT18 (MDP0000768369).

## Supplementary information

Revised - Supplementary materials-clean.
