# Supplementary material for: The apple C2H2-type zinc finger transcription factor MdZAT10 positively regulates JA-induced leaf senescence by interacting with MdBT2
Source: Hortic Res. 2021 Jul 1;8:159. doi: 10.1038/s41438-021-00593-0 (PMC8245655; doi:10.1038/s41438-021-00593-0)
Supplement: Supplementary file 1 — Revised - Supplementary materials-clean. [file 41438_2021_593_MOESM1_ESM.doc]

**Supplementary Figures**

**Figure S1.** Phylogenetic analysis and sequence analysis of plant ZAT10 proteins. **a** MdZAT10 and other 20 plants species ZAT10 proteins sequences were obtained from the NCBI database through the BLAST program. Phylogenetic analysis was performed via MEGA 5.2 software according to the amino acid sequences. The MdZAT10 protein was marked with a red asterisk. AtZAT10: *Arabidopsis thaliana*, AT1G27730; AhZAT10: *Arachis hypogaea*, XP_025613536.1; CsZAT10: *Cannabis sativa*, XP_030497975.1; CmZAT10: *Cucumis melo*, XP_008452639.1; DzZAT10: *Durio zibethinus*, XP_022766452.1; GrZAT10: *Gossypium raimondii*, XP_012445908.1; HsZAT10: *Hibiscus syriacus*, KAE8657056.1; JrZAT10: *Juglans regia*, XP_018852126.1; MrZAT10: *Morella rubra*, KAB1204032.1; MnZAT10: *Morus notabilis*, XP_024018014.1; PeZAT10: *Populus euphratica*, XP_011017126.1; PaZAT10: *Prunus avium*, XP_021832612.1; PdZAT10: *Prunus dulcis*, XP_034199820.1; PmZAT10: *Prunus mume*, XP_008220316.1; PpZAT10: *Prunus persica*, XP_007222874.1; PbZAT10: *Pyrus bretschneideri*, XP_009354104; RaZAT10: *Rhodamnia argentea*, XP_030537987.1; RcZAT10: *Rosa chinensis*, XP_024165704.1; TcZAT10: *Theobroma cacao*, XP_007018525.2; ZjZAT10: *Ziziphus jujuba*, XP_015885055.1. **b** The conserved domains of the ZAT10 proteins in 21 plants species. The sequences showed a highly conserved two zinc finger domains and an EAR motif.

**
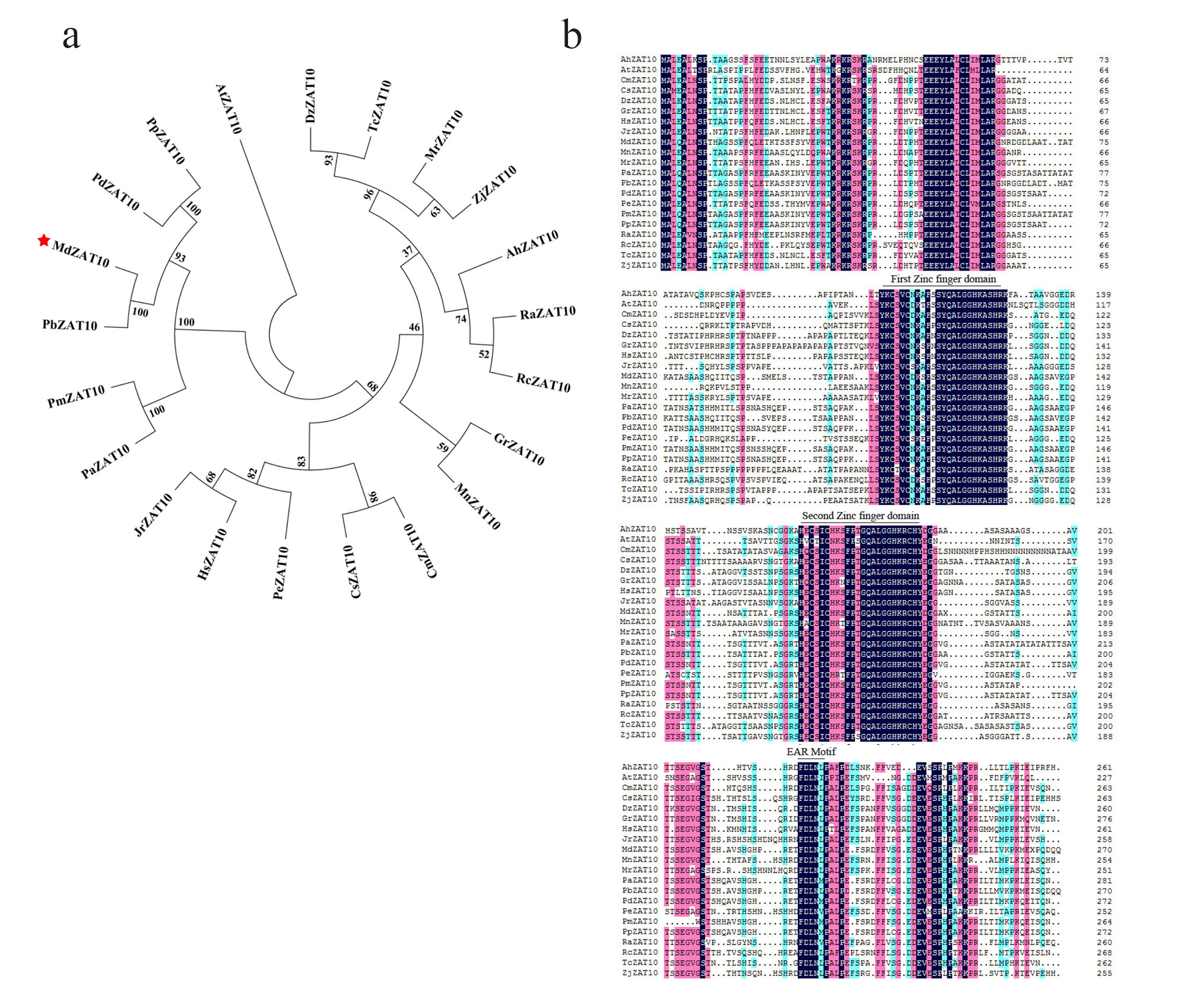
**

**Figure S2.** Identification of the transgenic plant materials. **a-c** The expression level of *MdABI5* (*MdABI5*-*L1*, *L2* and *L3*), *MdZAT10* (*MdZAT10*-*L1*, *L2* and *L3*) and *MdBT2* (*MdBT2*-*L1*, *L2* and *L3*) in ectopic transgenic *Arabidopsis* were analyzed by qRT-PCR. Col: wild-type *Arabidopsis*. **d-f** The expression level of *MdABI5*, *MdZAT10* and *MdBT2* in transgenic apple calli were analyzed by qRT-PCR. WT: wild-type apple calli; *MdABI5-OX*: *MdABI5* overexpressing apple calli; *MdABI5-Anti*: *MdABI5* antisense suppressing apple calli; *MdZAT10-OX*: *MdZAT10* overexpressing apple calli; *MdZAT10-Anti*: *MdZAT10* antisense suppressing apple calli; *MdZAT10-OX /MdABI5-OX*: overexpression of *MdZAT10* in the background of *MdABI5-OX* transgenic apple calli; *MdBT2-OX*: *MdBT2* overexpressing apple calli; *MdBT2-Anti*: *MdBT2* antisense suppressing apple calli; *MdZAT10-OX/MdBT2-OX*: overexpression of *MdBT2* in the background of *MdZAT10*-*OX* transgenic apple calli; **g-i** The expression level of *MdABI5* and *MdZAT10* in transiently transgenic apple leaves were analyzed by qRT-PCR. EV: empty vector; *MdABI5-OE*: *MdABI5* transiently transgenic apple leaves; *MdABI5-Anti*: *MdABI5* antisense suppressing transiently transgenic apple leaves; *MdZAT10-OE*: *MdZAT10* transiently transgenic apple leaves; *MdZAT10-Anti*: *MdZAT10* antisense suppressing transiently transgenic apple leaves; *MdZAT10-OE/MdABI5-OE*: *MdbZAT10* and *MdABI5* transiently co-transgenic apple leaves. *MdZAT10-OE/MdBT2-OE*: *MdZAT10* and *MdBT2* transiently co-transgenic apple leaves. **j** The expression level of *MdBT2* in transgenic apple seedlings were analyzed by qRT-PCR. GL-3: wild-type apple seedlings; *MdBT2-OE-L1/MdBT2-OE-L5*: *MdBT2* overexpressing apple seedlings; *MdBT2-Anti-L13/MdBT2-Anti-L23*: *MdBT2* antisense suppressing apple seedlings.


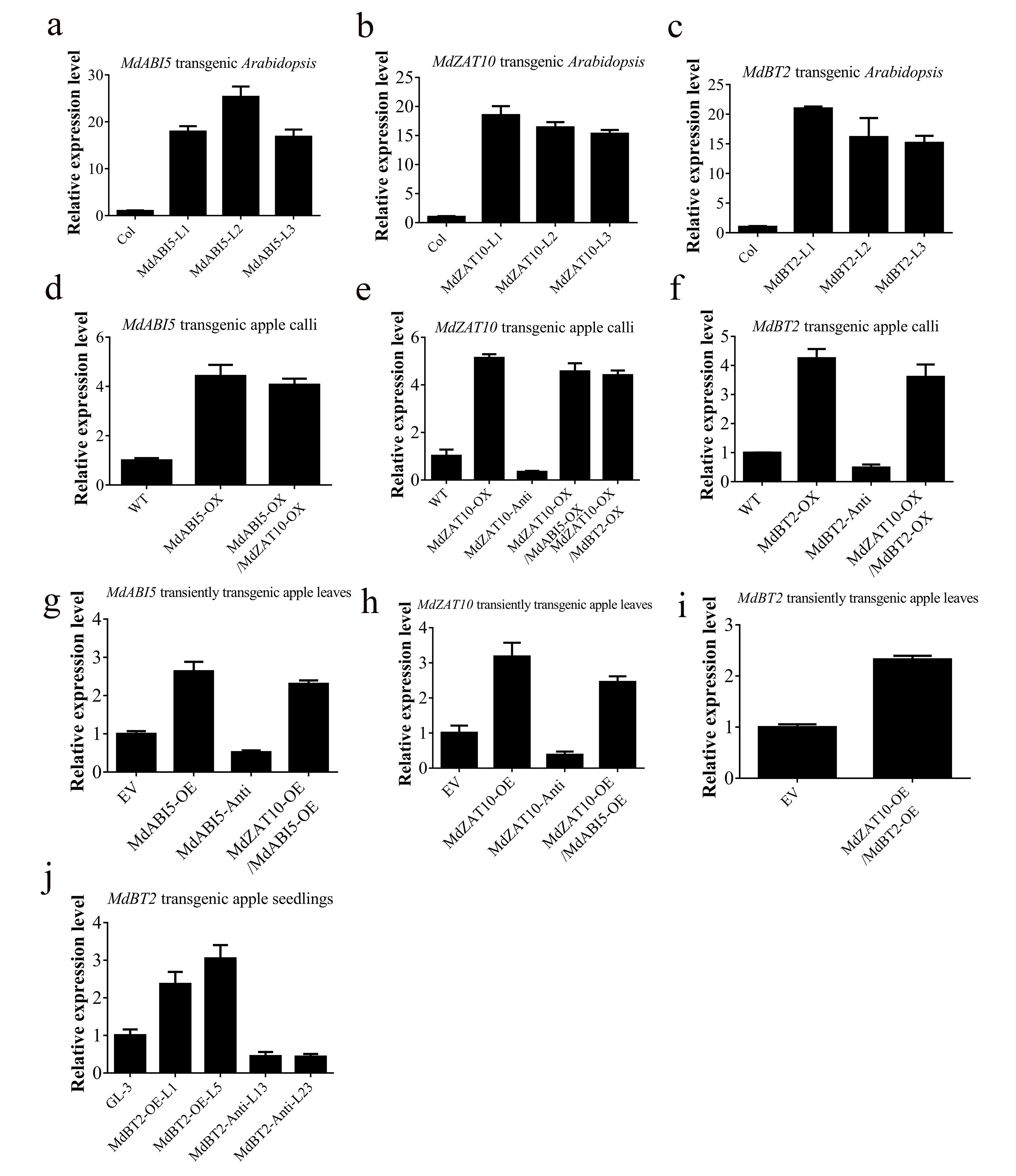


**Figure S3.** MdABI5 positively regulated leaf senescence. **a** Expression level of *MdABI5* in non-senescent (NS), early-senescent (ES) and late-senescent (LS). **b** Detached leaves of 3-week-old from Col and three transgenic lines (*MdABI5*-*L1*, *L2* and *L3*) were incubated on 3 mM MES buffer in the dark for 4 days. **c** Total chlorophyll content and **d** *Fv/Fm* ratio was determined before and after dark treatment. **e** Leaf senescence phenotype and **f** total chlorophyll content of transiently expressing apple leaves of empty vector (EV), *MdABI5*-overexpressing and *MdABI5* antisense in the dark for 16 d. **g-h** The expressions level of *MdNYC1* and *MdNYE1* in apple leaves after 16d dark treatment. Asterisks indicate significant differences by *t*-test (**P < 0.05, **P < 0.01*).


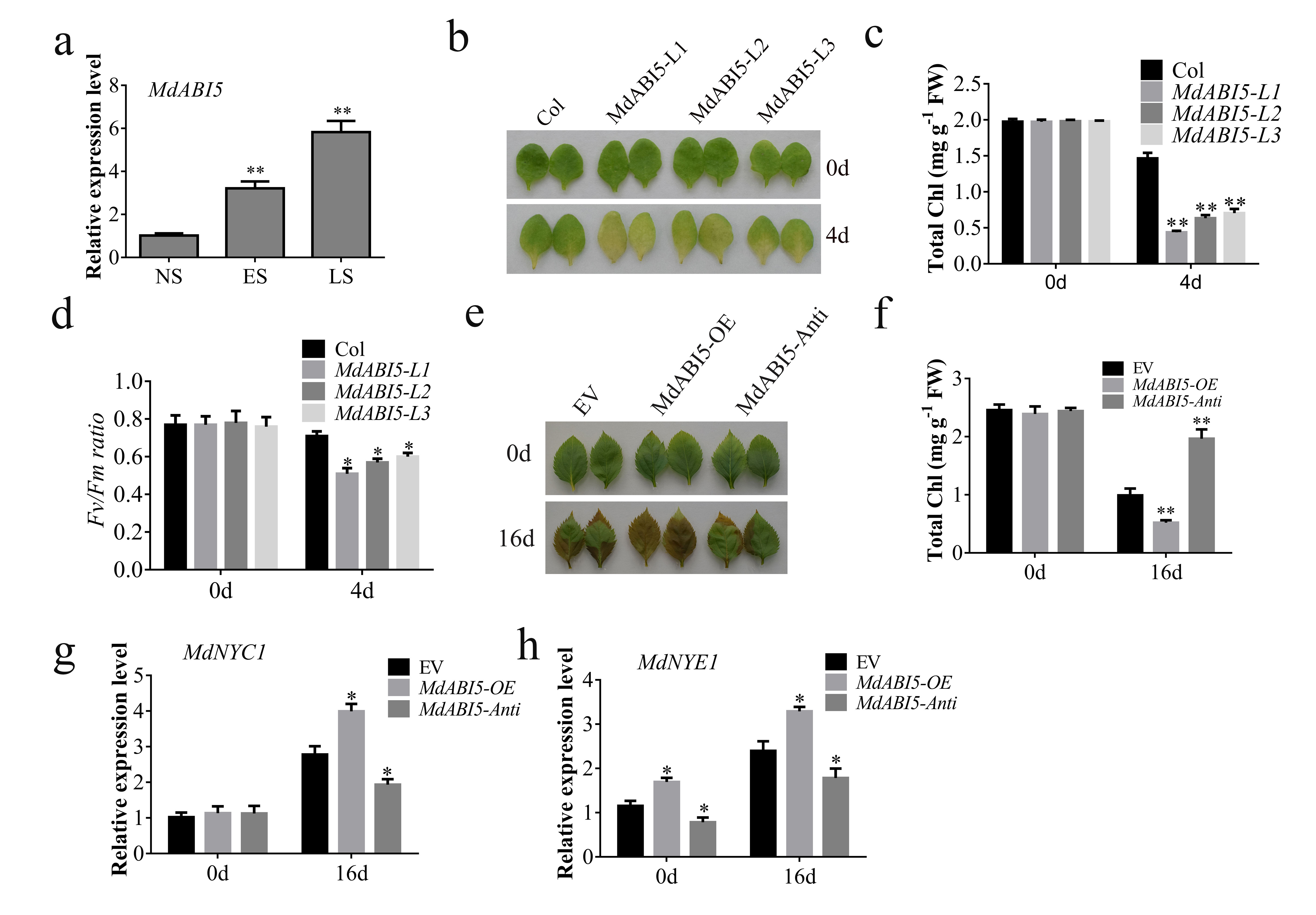


**Figure S4.** Effects of MeJA on the protein stability and protein level of MdZAT10. **a** Degradation of the MdZAT10-His protein and its stabilization by MeJA or MG132 *in vitro*. **b** MdZAT10 protein level analysis of overexpressing *MdZAT10* transgenic apple calli with MeJA treatment.

**
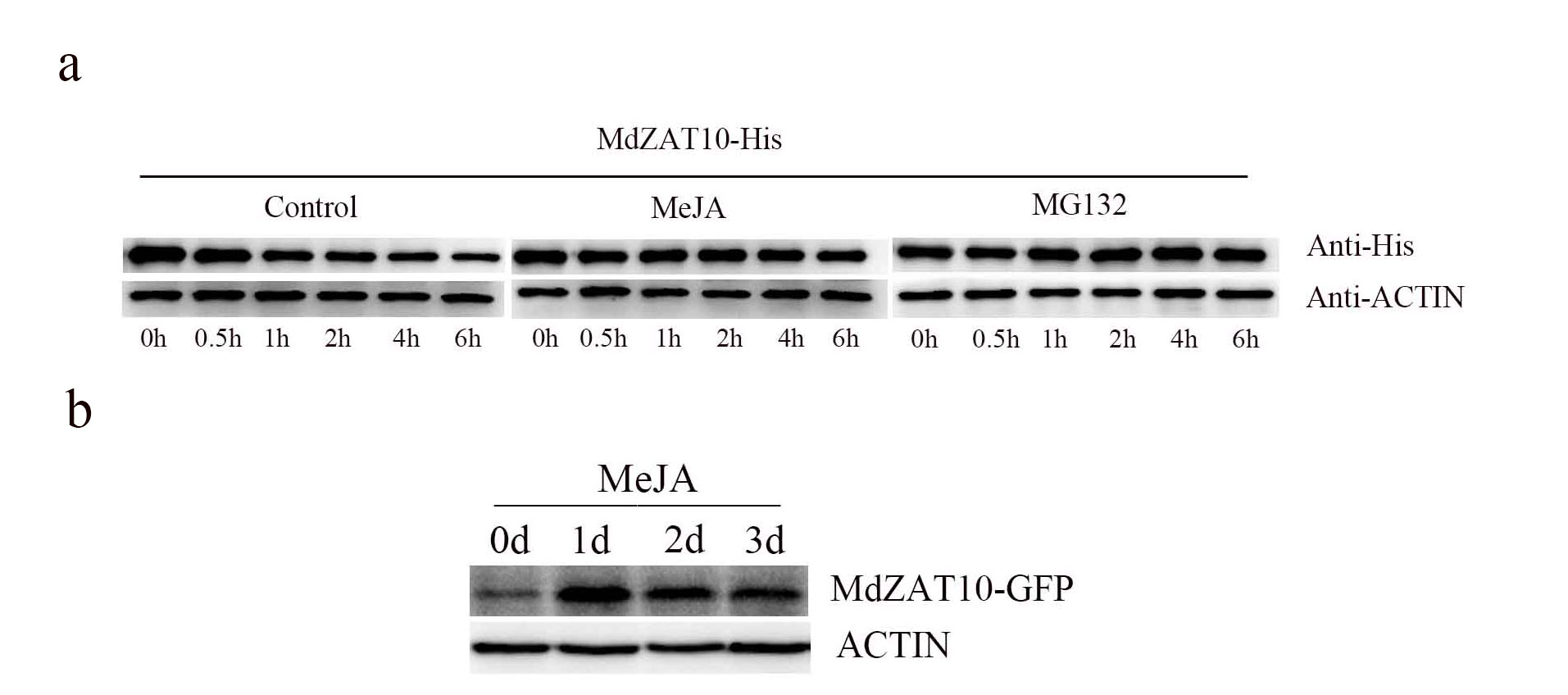
**

**Figure S5.** Extensive interactions between MdBTs and MdZATs. **a** Y2H assay showing the interaction between MdBT2 with MdZATs proteins. **b** Y2H assay showing the interaction between MdBTs with MdZAT10 protein (MdBT1:MDP0000151000, MdBT2: MDP0000643281, MdBT3.1: MDP0000296225, MdBT4: MDP0000215415).

**
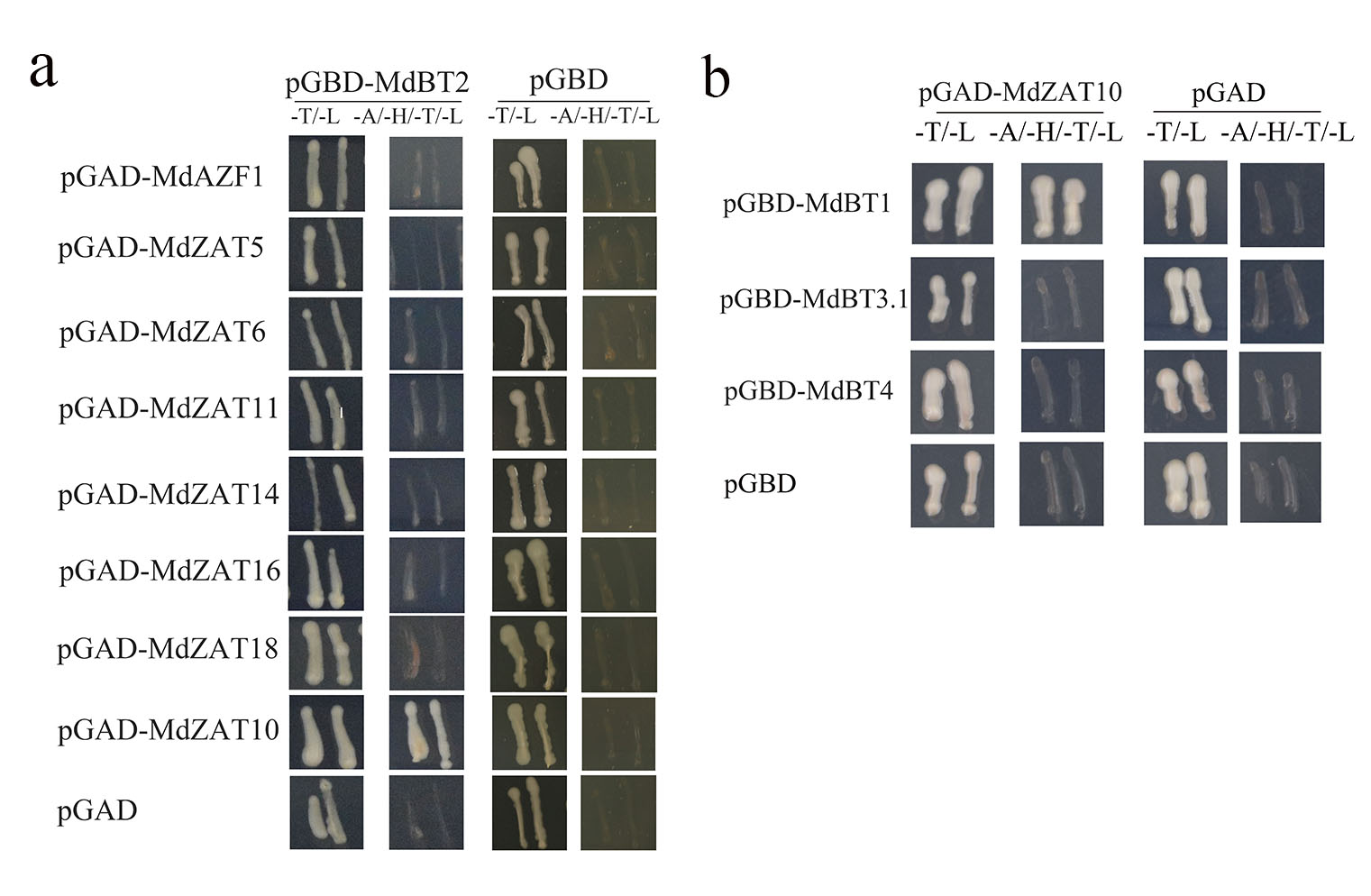
**

**Figure S6.** Overexpression of *MdBT2* delays JA-induced senescence in *Arabidopsis* leaves. **a** Leaf senescence phenotype and **b** chlorophyll content of detached leaves from *Arabidopsis* of Col and *MdBT2-L1, L2 and L3* were floated on 3 mM MES buffer with 100 μM MeJA in dark for 5d.

**
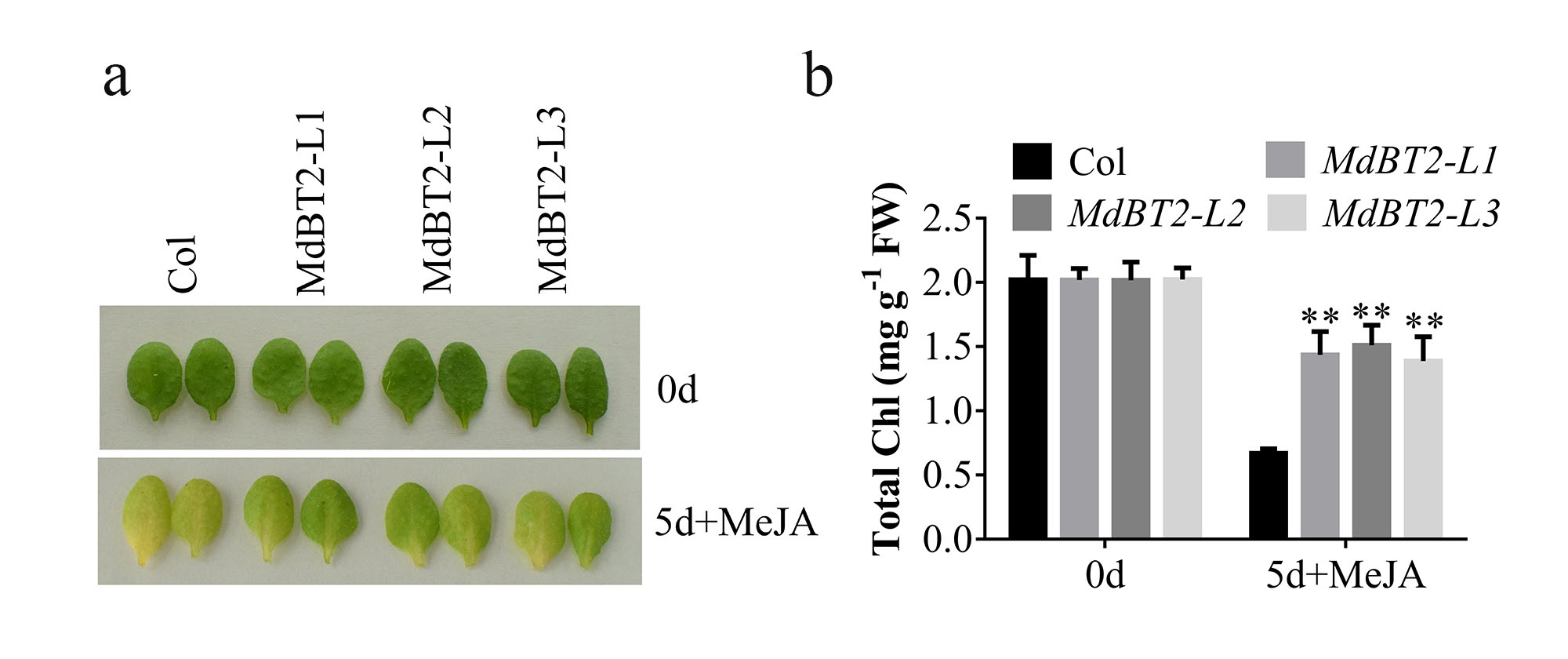
**

**Figure S7.** Analysis the expression level of senescence-related genes, JA biosynthesis pathway genes and other genes. qRT-PCR analysis of expression level of senescence-related genes (*MdPAO* and *MdSAG29*), JA biosynthesis pathway genes (*MdAOC1* and *MdAOS*), *MdWRKY70* and *MdAPX2* in apple calli of wild-type (WT), *MdZAT10* overexpression (*MdZAT10-OX*) and antisense *MdZAT10* (*MdZAT10-Anti*). Asterisks indicate significant differences by *t*-test (**P < 0.05*).


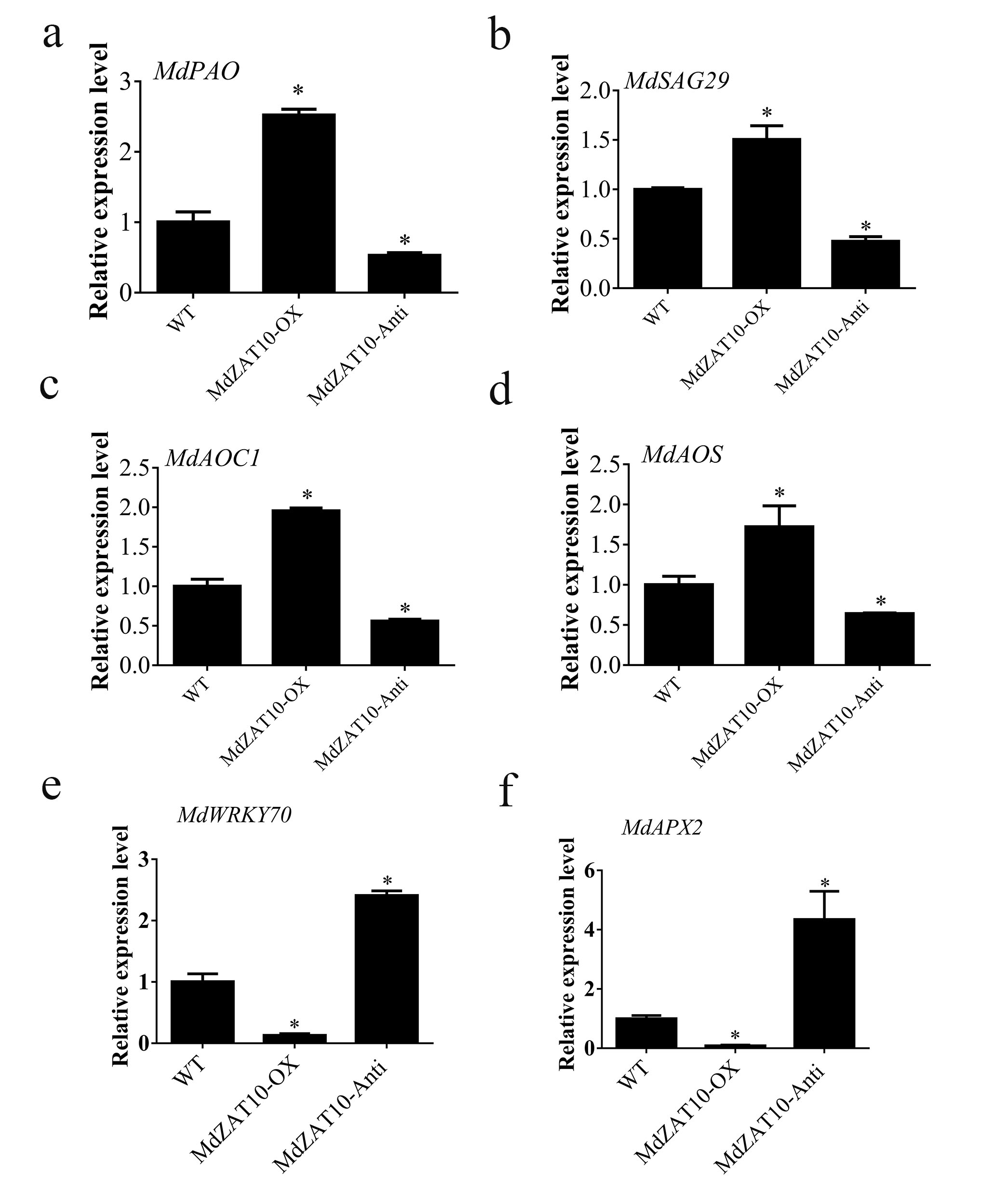


**Supplementary Tables**
**Supplementary Table 1**. Primers used for gene cloning

| Primer name | sequence (5’ to 3’) |
| --- | --- |
| MdZAT10-F | ATGGCTCTGCAAGCTCTC |
| MdZAT10-R | ATTTTGTTGGTCCTGTGGA |
| MdZAT10-Anti-F | TGTTGACGTGGAGAGCTC |
| MdZAT10-Anti-R | ATGGCTCTGCAAGCTCTCA |
| MdZAT10-N-AD-F | ATGGCTCTGCAAGCTCTCAA |
| MdZAT10-N-AD-R | CCGACTCCCTCAGAGGAAGTTATA |
| MdZAT10-C-AD-F | GGATCCACCGCTACCACA |
| MdZAT10-C-AD-R | ATTTTGTTGGTCCTGTGGA |
| MdAZF1(AD)-F | GAATTCATGGCCTTAGAGGCTTTGAAA |
| MdAZF1(AD)-R | GTCGACATCTCCACCCAACAACCAC |
| MdZAT5(AD)-F | GAATTCATGGAAGGCCAAGAAGAACTAG |
| MdZAT5(AD)-R | GTCGACATAATGACAATCCACCAAAGCAG |
| MdZAT6(AD)-F | GAATTCATGGCCTTAGAGGCTTTGAA |
| MdZAT6(AD)-R | GTCGACATCTCGACCCAACAACCAC |
| MdZAT11(AD)-F | GAATTCATGAAGAGAGATATGAGAGCAG |
| MdZAT11(AD)-R | GTCGACTGATGCAAAAGCATCAACATTTG |
| MdZAT14(AD)-F | GAATTCATGATGATGATGGAAGCTGCAG |
| MdZAT14(AD)-R | GTCGACGTATTGGCAACCCACCAAAG |
| MdZAT16(AD)-F | GAATTCATGAACATGAAGAGAAGCATCACAG |
| MdZAT16(AD)-R | GTCGACCAAAAACCCAACAAGTGGAGC |
| MdZAT18(AD)-F | GAATTCATGAAGAGAGATATGAGAGGAGAA |
| MdZAT18(AD)-R | GTCGACTGATACAAAAGCATCAACTTTTGGT |
| MdABI5-F | ATGGGTGTTTCGGAGTCGGAAATC |
| MdABI5-R | CATAATCTTCACTTGCAGTGGAG |
| MdABI5-Anti-F | TGGTTTGGAAATGGGTGGACT |
| MdABI5-Anti-R | TCGAGCTCTGCCAGCGCCTGA |
| MdABI1(BD)-F | GAATTCATGGAGGATATGTCTCCGGTG |
| MdABI1(BD)-R | GTCGACTGTTTTGCTTTTAAACTTCCTTTG |
| MdABI2(BD)-F | GAATTCATGGAGGATATGTCTACGCCG |
| MdABI2(BD)-R | GTCGACACGTTTTGCTTTTAAACTTCCTTTG |
| MdABI4(BD)-F | GAATTCATGGAAGAAGACCAACACCG |
| MdABI4(BD)-R | GTCGACTGAATCCAATCCCTTAAAATCCA |
| MdBT2-F | ATGGAAGCTAATCCGACCGCAAC |
| MdBT2-R | CAATCTGAAGCTTCTAATTCCA |
| MdBT2-Anti-F | ATTCTTGCAAGCTCACGACCCTTG |
| MdBT2-Anti-R | ATCTGGAGGCCTTGACACGTG |
| MdBT1 (BD)-F  MdBT1 (BD)-R | CGTCGACAGTTATACTTGCTCCTGG  CAGATCTCACGTCCACAATGAACATG |
| MdBT3.1 (BD)-F | ATGGCTTCATCTACTCCGG |
| MdBT3.1 (BD)-R | TCATGATAAACGCGAGTGG |
| MdBT4 (BD)-F | ATGTGTAAGGTGAAAAACAT |
| MdBT4 (BD)-R | TCACTGCCACAAAGTGCTG |

**Supplementary Table 2**. Primers used for qRT-PCR analysis of different genes

| Primer name | sequence (5’ to 3’) |
| --- | --- |
| MdZAT10-F | GGTCAACTTCTCACGCGGTCAG |
| MdZAT10-R | GGCTCTCCACCTCCTCCTCAC |
| MdABI5-F | AGTCCTCTGCGTATGCTGCGAATG |
| MdABI5-R | ATCATCCTTCTCTGCCTCCTC |
| MdBT2-F | GTTGAGGGAGATAGGGTTTGAG |
| MdBT2-R | GGTAAATGTAAAACAATAGAGAGG |
| MdNYC1-F | ATCACTTTTAAAGGAGTGCAAGCG |
| MdNYC1-R | TGTCAAGTAATTGATGGCCTTTCC |
| MdNYE1-F | ACTTCATCTTCTGCAAAGAATTAC |
| MdNYE1-R | TGGTGGTGGGCACCGCCTGATGAC |
| MdSAG29-F | ATCCGAACAAGAAGTGTCGAAT |
| MdSAG29-R | ATCTTCTATTATCACCTGCT |
| MdPAO-F | AGCACCTTGCTATTATTTGAAT |
| MdPAO-R | GCCACCAAGCGGGCCCTGGCAT |
| MdWRKY70-F | CAGAACCCGTACGGAGATCG |
| MdWRKY70-R | TGGCCATCAGAAGCAGTCAG |
| MdAPX2-F | GCTGGCTGGAGTTGTTGCTGTAG |
| MdAPX2-R | CGACCTTCTGGTGGTGGTTCTTG |
| MdAOC1-F | CTCGATCAGGTCTCCTCCTCCATC |
| MdAOC1-R | ATGCGGATTTAAGGGTTGGGAAGG |
| MdAOS-F | AACCAGGGTAGGGACGAGTTCTTC |
| MdAOS-R | GGGCGACGACCTTGGATTTGTAG |
| 18S-F | ACACGGGGAGGTAGTGACAA |
| 18S-R | CCTCCAATGGATCCTCGTTA |

**Supplementary Table 3.** List of the abbreviation.

| Abbreviations | Full name |
| --- | --- |
| AD | pGAD424 |
| Anti | antisense suppression |
| AZF2 | Arabidopsis zinc-finger protein 2 |
| BD | pGBT9 |
| Chl | chlorophyll |
| EAR | ERF-associated amphiphilic repression |
| ES | early-senescent |
| IPTG | isopropyl β-D-1-thiogalactopyranoside |
| JA | jasmonic acid |
| LS | late-senescent |
| MeJA | methyl jasmonate |
| NYC1 | NON-YELLOW COLORING1 |
| NYE1 | STAY-GREEN 1 |
| NS | non-senescent |
| OE | overexpression in apple seedling |
| OX | overexpression in apple calli |
| PAO | pheophorbide a oxygenase |
| SAG | senescence-associated gene |
| STZ/ZAT10 | SALT TOLERANCE ZINC FINGER |
| TF | transcription factor |
| ZFPs | zinc finger proteins |
